# Ambipolar Superconductivity with Strong Pairing Interaction in Monolayer 1T′-MoTe_2_

**DOI:** 10.1021/acs.nanolett.3c02033

**Published:** 2023-08-04

**Authors:** Fangdong Tang, Peipei Wang, Qixing Wang, Yuan Gan, Jian Lyu, Xinrun Mi, Mingquan He, Liyuan Zhang, Jurgen H. Smet

**Affiliations:** † Max Planck Institute for Solid State Research, Stuttgart 70569, Germany; ‡ Department of Physics and Shenzhen Institute for Quantum Science and Engineering, Southern University of Science and Technology, Shenzhen 518055, China; § Low Temperature Physics Laboratory, College of Physics & Center of Quantum Materials and Devices, Chongqing University, Chongqing 401331, China

**Keywords:** ambipolar superconductivity, strong pairing interaction, monolayer 1T′-MoTe_2_, gate tunability, intrinsic bandgap

## Abstract

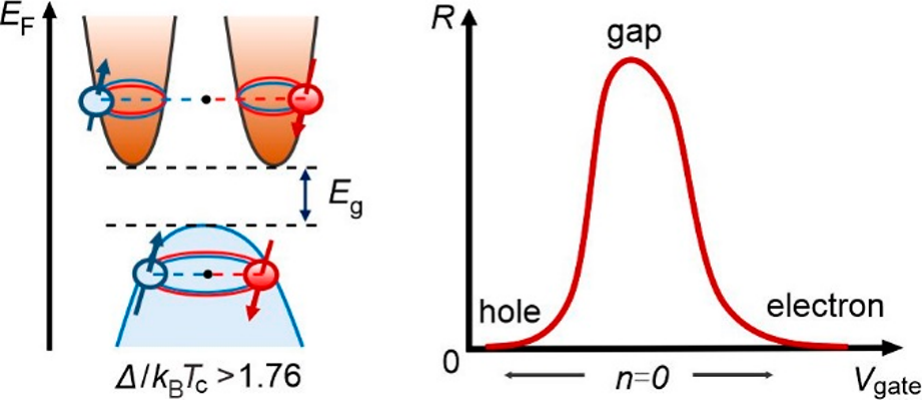

Gate tunable two-dimensional (2D) superconductors offer significant advantages in studying superconducting phase transitions. Here, we address superconductivity in exfoliated 1T′-MoTe_2_ monolayers with an intrinsic band gap of ∼7.3 meV using field effect doping. Despite large differences in the dispersion of the conduction and valence bands, superconductivity can be achieved easily for both electrons and holes. The onset of superconductivity occurs near 7–8 K for both charge carrier types. This temperature is much higher than that in bulk samples. Also the in-plane upper critical field is strongly enhanced and exceeds the BCS Pauli limit in both cases. Gap information is extracted using point-contact spectroscopy. The gap ratio exceeds multiple times the value expected for BCS weak-coupling. All of these observations suggest a strong enhancement of the pairing interaction.

Gate tunable 2D superconductors allow for a systematic study of the superconducting phase transition as a function of density without the need of producing multiple samples with different compositions. While in the majority of cases either the very potent ionic liquid gating technique^1,2^ or the proximity effect^3,4^ is required to induce superconductivity, there are also a few materials that exhibit intrinsic superconductivity at low densities that can be more conveniently reached with field effect gating. Reported examples include oxide heterostructure interfaces,^5,6^ twisted bilayer graphene,^7,8^ and 1T′-WTe_2_ monolayers.^9,10^ The last is a member of the family of transition metal dicalchogenides (TMDs MX_2_, with M = Mo or W and X = S, Se, or Te) that come in different phases. Recently, the 1T′-phase and the *T*_d_-phase have been intensively investigated.^11,12^ They are predicted to be type-II Weyl semimetals in bulk form^13,14^ and large-gap 2D topological insulators (TI) in monolayer form.^15^ Band gap opening in the monolayer was predicted, initially as the combined result of band inversion and spin–orbit coupling (SOC)^15−17^ and later also other mechanisms were put forward^18,19^ (see also Section 6, Supporting Information (SI)). In the remainder, we focus on the 1T′-MoTe_2_ phase. Different from the bulk system with a superconducting transition temperature of 0.1 K,^12^ the critical temperature is significantly enhanced in few-layer samples.^20−22^ This behavior with thickness is rare in 2D superconductors. While several studies have observed insulating behavior in 1T′-MoTe_2_ monolayers_,_ which they attributed either to excessive disorder due to oxidation^23^ or the formation of a gap in the bulk,^11^ other works have claimed semimetallic behavior and the absence of a gap in the bulk instead.^16,17,22^ Disorder and sample degradation during handling^23,24^ may be responsible for these contradictory results. Hence, continued efforts to unveil the intrinsic physics of the monolayers are desirable. In this work, we provide evidence that 1T′-MoTe_2_ monolayers are insulating. We also demonstrate ambipolar superconductivity for electrons in the Q-valleys and holes near the Γ-point in the valence band. The onset of superconductivity increases all the way up to ∼7.5 K, and the in-plane critical fields *B*_*c*__2,∥_ exceed the BCS Pauli limit.^25,26^ This enhanced stability of the superconductivity is attributed to stronger pairing interactions.

Measurements were performed on 1T′-MoTe_2_ devices fabricated from single crystals (Method Section, SI). Here we discuss monolayer devices (D1–D5) that exhibit insulating behavior at low density. An exceptional noninsulating device (D6) is discussed in Section 13 of the SI. Devices D2–D5 have both a back-gate (doped Si substrate) as well as a top-gate. The latter consists of a graphitic layer on top of encapsulating hBN. A device schematic is illustrated in Figure 1A. Optical images are shown in Figure S1 (SI). The extraction of the carrier density is described in Section 3 in the SI. Figure 1B schematically depicts the band structure of monolayer and multilayer 1T′-MoTe_2_.^11,15−17,21−23^ Band inversion arises from the period doubling of the Mo chain in the 1T′ structure. It lowers the Mo d-orbital below the Te p-orbital near Γ. It has been argued that the presence of SOC can open a topological band gap in the monolayer.^15−17^ Also the strong on-site Coulomb interaction in Mo^16,27^ and a potential excitonic instability^18,19^ may play a role for the formation of a gap. Assessing the relative importance of these different mechanisms that influence the formation of a gap is difficult and may account for the uncertainty and variation of the different electronic phases that have been reported in experiments^11,16,17,22^ (Section 6, SI). The temperature dependence of the sheet resistivity *ρ*_*s*_ for samples of different thicknesses is summarized in Figure 1C (see also Section 2, SI). *ρ*_*s*_ increases significantly from four layers to one layer. The temperature at which *ρ*_*s*_ starts to drop has been marked in the inset. It systematically decreases with increasing thickness. In the remainder, 50% of the normal state resistance will be used as the criterion for the transition temperature, referred to as *T*_*c*__,0_ in the absence and *T*_*c*_ in the presence of a magnetic field. For thicknesses above two layers, the samples display metallic behavior and *T*_*c*__,0_ < 3 K. For monolayer devices, *ρ*_*s*_(*T*) depends on the density. At high density, samples behave metallic across the entire temperature range and turn superconducting below 7.5 K. For low density, samples undergo a metal–insulator transition (MIT). This heralds the opening of a band gap. The resistance drops sharply near 7.5 K signaling the superconducting transition. Such a low-density case is illustrated in Figure 1C. When the density is reduced further, the band gap fully develops and the sample remains insulating down to the lowest temperature (Figure 2B, device D4 in Figure S4C, SI). The gap size is estimated from an Arrhenius fit (Section 6, SI) to be ∼7.3 meV. Above 100 K (∼8.7 meV), thermally activated charge carriers dominate the transport. In this regime, reduced phonon scattering with decreasing temperature produces a metallic behavior. The MIT appears below 100 K as the bulk gap opens. The shift to higher temperature of the onset of superconductivity (arrows in the inset Figure 1C) is consistent with previously reported results on few layer and monolayer devices.^20,22^ Since theoretical calculations on 1T′-MoTe_2_ monolayers predict single band behavior without any signature of a density-wave induced modulation of the density of states, this temperature enhancement is most likely a consequence of increased pairing interactions.^22,28,29^ This will be elaborated upon below.

**Figure 1:**
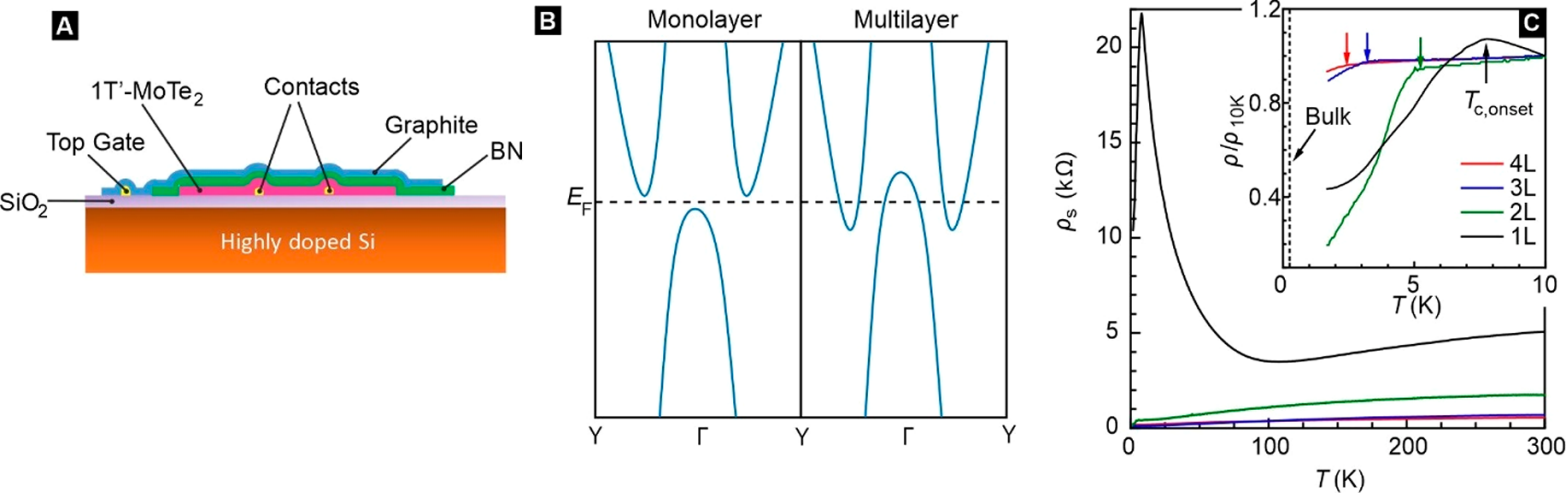
1T′-MoTe_**2**_ device geometry and basic transport characterization. (A) Cross-sectional view of the device structure. (B) Schematic of the band structure for monolayer (left) and multilayer (right) 1T′-MoTe_2_. (C) Temperature dependence of the sheet resistivity for samples with different layer thickness from 1 (1L) to 4 layers (4L). The inset shows a zoom of the sheet resistivity normalized to its value at 10 K. Arrows mark the temperature where the resistance starts to drop. This signals the onset of superconductivity. The temperature is referred to as *T*_*c*__,*onset*_.

**Figure 2:**
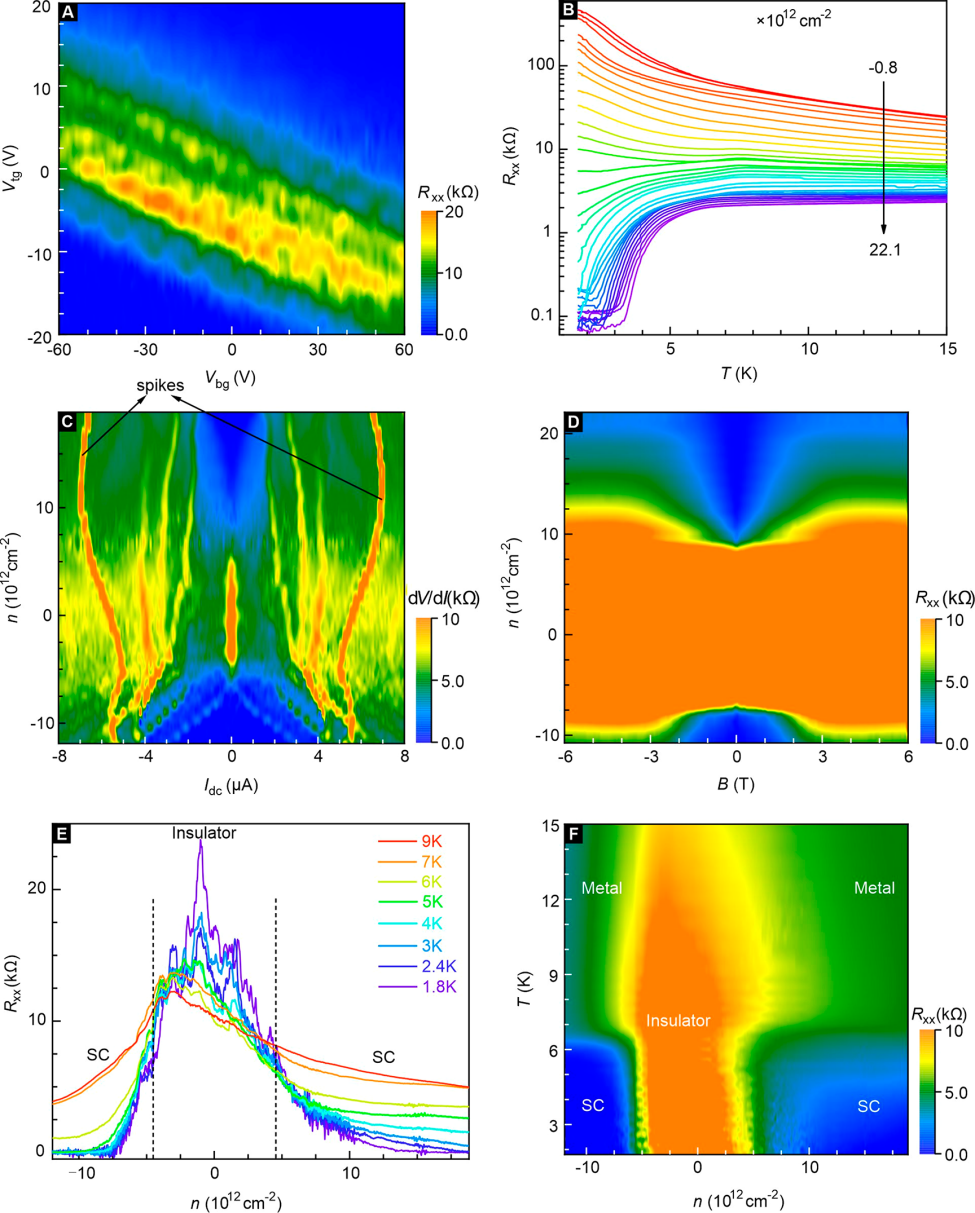
Longitudinal resistance and differential resistance as a function of the density, temperature and magnetic field. (A) Resistance measured at *T* = 1.8 K and *B* = 0 T as a function of the top and bottom gate voltages for monolayer device D3. (B) Temperature-dependent resistance at zero magnetic field with density as an additional discrete parameter. The transition from an insulating state to n-type superconductivity is clearly visible. Data were recorded on device D4. (C) Color map of the differential resistance d*V*/d*I* in the parameter plane spanned by the dc bias current and the carrier density. Data were taken on device D3 at *T* = 1.8 K and *B* = 0 T. (D) Color map of the magnetoresistance for a perpendicular magnetic field and as a function of the carrier density. Data were taken on device D4 at *T* = 1.7 K. (E) Longitudinal resistance as a function of the density for different temperatures ranging from 1.8 to 9 K. The dashed black lines roughly mark the boundary between the insulating phase and the superconducting states. Data were taken on device D3 at *B* = 0 T. (F) Color map of the longitudinal resistance in the temperature vs density parameter plane. The different states are marked. Single line traces from this data set are shown in panel (E).

We intend to compile a diagram of the different electronic states that emerge as the density is tuned. Figure 2A displays a 2D color map of the gate-dependent resistance for device D3 at 1.8 K and a zero magnetic field. As the density is tuned, the sample undergoes a transition from a p-type (around the Γ-point) superconducting state to an insulating state with a gap inside the bulk and finally a n-type superconducting state (around the Q-point). The superconducting behavior is observed for electron and hole densities above 7 × 10^12^ cm^–2^. This contrasts with 1T′-WTe_2_ monolayers where superconductivity only appears for electrons.^9,10^ Data for two additional devices D2 and D4 can be found in Section 3, SI. In the insulating state, Hall measurements are problematic and the carrier density is only a rough estimate.^9,22^ Both charge carrier types are likely simultaneously present due to inhomogeneous disorder. Figure 2B illustrates the evolution of the temperature-dependent resistance for the monolayer sample D4. For a low net hole doping of −8 × 10^11^ cm^–2^ clear insulating behavior is observed. This behavior also persists for low net electron doping, but the resistance decreases with increasing electron density. Eventually the system behaves metallicly, and at sufficiently high electron density, superconducting behavior appears. It is very pronounced at 2.21 × 10^13^ cm^–2^, the highest density shown. Additional data for other devices are displayed in Section 4, SI. For the data recorded on device D4 in Figure 2B, the resistance remains nonzero down to the lowest temperature at the highest accessible density, despite the clear superconducting transition. This is a clear case of a so-called “failed superconductor” also referred to in the literature as an anomalous metal or a quantum metal. Its transport changed with density and magnetic field and also the degree of disorder.^2,30,31^ These systems typically exhibit quantum critical behavior with a magnetic field. Superconducting regions are rare (Section 11 of SI),^31,32^ and quantum fluctuations play an important role in this inhomogeneous superconductor.

Differential resistance data recorded in the dc bias current versus density parameter plane are also helpful to identify transitions. An example is shown in Figure 2C. This color map of the differential resistance was acquired on monolayer D3 at 1.8 K. Additional data sets are available in Section 6 of the SI. For low |*n*| ≲ 7 × 10^12^ cm^–2^, a sharp peak is observed in the differential resistance at low bias current. It signals the insulating ground state. As the carrier density is increased beyond |*n*| ≥ 7 × 10^12^ cm^–2^, the amplitude of this zero-bias peak gradually drops. When entering the superconducting regime, the differential resistance remains zero. The region of zero differential resistance becomes wider as the superconducting ground state becomes better developed at larger densities. For larger bias currents, additional differential resistance peaks are observed. The bias currents at which they appear vary in a continuous but erratic manner on density. This may be related to a rearrangement of the insulating and superconducting regions with density (Section 5, SI). Figure 2D illustrates the density dependence of the magnetoresistance recorded on device D4 in a perpendicular magnetic field at 1.7 K. Here, the insulating state and superconducting states can be distinguished better because the resistance value changes more dramatically in the three different regimes. The out-of-plane critical field *B*_*c*__2,⊥_ at higher densities is ∼2 T (Figure S11B and Table S1). This critical field is much larger than that of most low-density superconductors (∼0.1 T). Figure 2E plots the evolution of the resistance with temperature between 1.8 and 9 K at zero field for monolayer D3. The sequence of phase transitions from a p-doped superconducting state to an insulating state and from the insulating state to an n-doped superconducting state is visible (see Section 7, SI). There is no clear critical density *n*_*c*_ as observed in monolayer 1T′-WTe_2_.^9^ Instead, there is a transition region between 3× 10^12^ < |*n*| <7 × 10^12^ cm^–2^ that forms the phase boundary between the insulating and superconducting state. In the insulating regime the resistance typically exhibits strong fluctuations, and depending on the quality, values as large as ∼10^5^ ohms are observed in device D4 (see also Sections 7 and 8, SI). In the low-density region, insulating and superconducting regions coexist. The latter are linked by Josephson-like coupling that varies with the density.

A diagram summarizing the different phases detected in device D3 is displayed in Figure 2F. For other samples we refer to Section 4 of the SI. In sample D3 the insulating state around zero average carrier density persists up to |*n*| < 3 × 10^12^ cm^–2^ with superconducting states on either side for electron and hole doping. They convert into normal metallic states above the transition temperature. For hole carriers, the transition to the superconducting state is much sharper and clearer than that for electrons. This difference between holes and electrons can be understood from the band structure. The effective mass and density of states are significantly larger for hole doping at the Γ-point than for electrons at the Q-points. The onset of superconductivity varies little with the average density, because Cooper pair formation occurs as long as *T* < *T*_c,onset_, since superconducting puddles even exist at low average density due to the density inhomogeneity.

In the following, we focus on the unconventional nature of the superconductivity. Figure 3A illustrates the temperature dependent resistance recorded on device D2 for different perpendicular (*B*_⊥_, left panel) and parallel fields (*B*_∥_, right panel) and a fixed density of 2.38× 10^13^ cm^–2^. The in-plane and out-of-plane upper critical fields are shown as functions of the normalized temperature in Figure 3B with blue circles (*B*_*c*__2,∥_) and black squares (*B*_*c*__2,⊥_). The temperature is normalized with *T*_*c*__,0_. The experimental data are fitted to the 2D Ginzburg–Landau (GL) model in order to extract the zero temperature Ginzburg–Landau in-plane coherence length *ξ*_*GL*_ using the expression . Here  is the magnetic flux quantum for Cooper pairs.^33^ A least-squares fit to the data points in Figure 3B yields *ξ*_*GL*_ (0K) ≈ 12.1 nm. The BCS coherence length ξ_0_ then follows from the relationship ξ_0_*=* 1.35*ξ*_*GL*_ (0K)^33,34^ giving ξ_0_ ≈ 16.3 nm. The mean free path of the charge carriers is estimated from the Drude model, , where *ρ*_*s*_ is the sheet resistivity, *n*_*e*_ is the net charge carrier density, and *g*_*s*_ = *g*_*v*_ = 2 for spin and valley degeneracy in the case of electrons.^10,22^ We obtain a mean free path at *l*_*mfp*_ ≈ 7.8 nm. Since ξ_0_ > *l*_*mfp*_, the superconductor is in the dirty limit. For the in-plane critical field, the phenomenological dependence of *B*_*c*__2,∥_ (*T*) ∝ (1 – *T*/*T*_*c*__,0_)^1/2^ is used to fit the data near *T*_*c*__,0_.^33,35^ Such a fit yields *B*_*c*__2,∥_ (0K) ≈ 19 ± 1 T. This value is about 2 times larger than the BCS Pauli limit *B*_*p*_^*BCS*^ (0K) = 1.85*T*_*c*__,0_ ≈ 8.7 T (horizontal black dashed line). This is consistent with previous reports.^20−22^ Similar behavior is observed for holes. In Figure 3D, data are shown for a hole density of −1.10 × 10^13^ cm^–2^. Here too, *B*_*c*__2,⊥_ (*T*) exhibits a linear temperature dependence as well as an enhanced *B*_*c*__2,∥_ (0K) compared to that of the BCS Pauli limit. The angular dependence of the upper critical field (*B*_*c*__2_ (θ)) at a temperature of 1.65 K is plotted in Figure 3C. The critical field is defined here as the field where the resistance reaches 35% of the normal state resistance. Data were fitted to the 2D Tinkham model (black line) as well as the 3D anisotropic Ginzburg–Landau model (blue line).^21,33^ The former model achieved better agreement. Also the divergent behavior of the *B*_*c*__2,∥_/*B*_*c*__2,⊥_ ratio when approaching *T*_*c*__,0_ plotted in the inset to Figure 3B corroborates the 2D nature of superconductivity.^36,37^ The key density dependent superconducting parameters are summarized in Section 9 of the SI (Figure S11).

**Figure 3:**
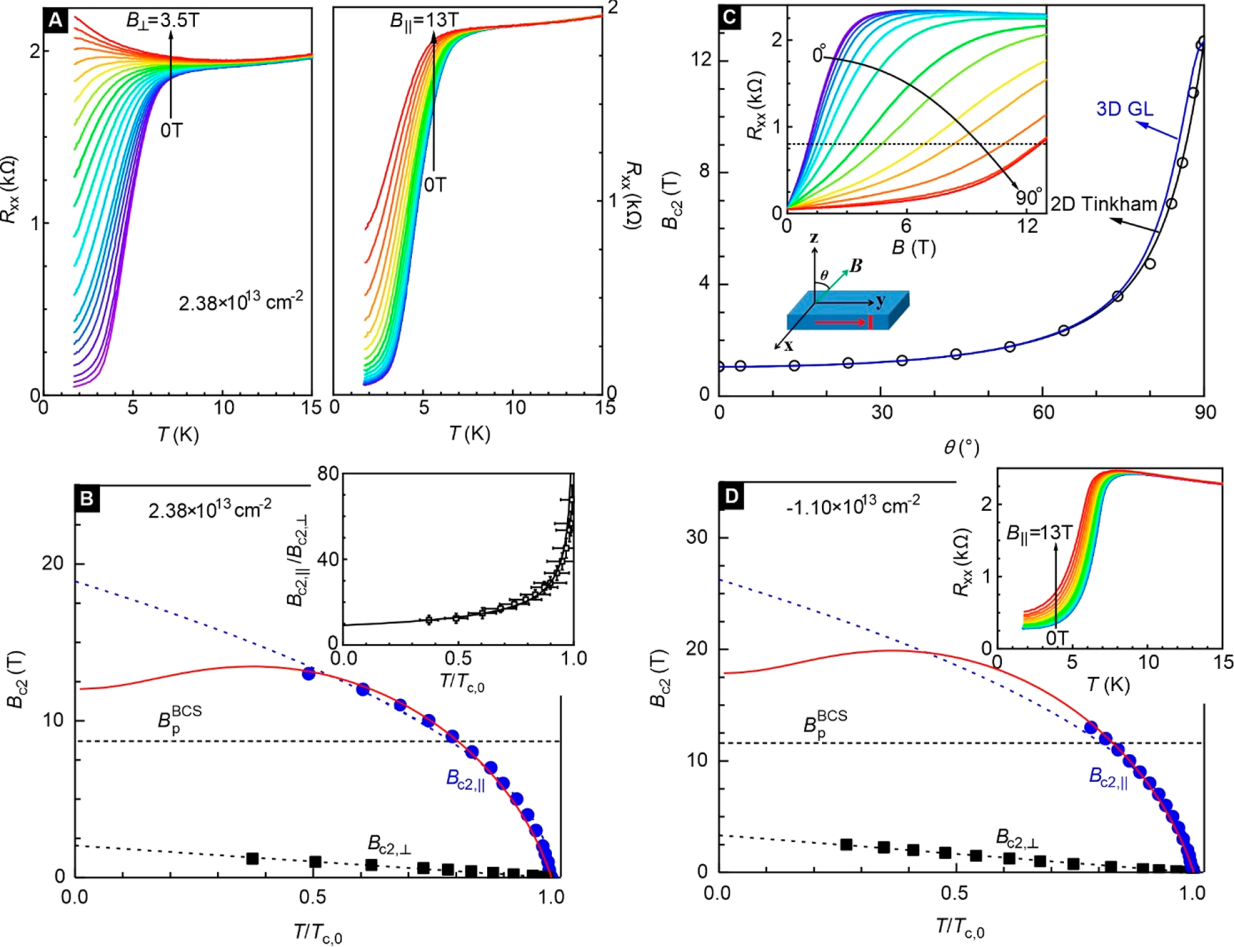
In-plane and out-of-plane magnetoresistance and critical field data. All data were recorded on device D2. (A) Temperature dependent magnetoresistance for different perpendicular magnetic fields *B*_⊥_ (left panel) and parallel magnetic fielda *B*_∥_ (right panel). The data are recorded for an electron density of 2.38 × 10^13^cm^–2^. (B) In-plane (black squares) and out-of-plane (blue circles) upper critical field as a function of temperature. The temperature is normalized to *T*_*c*__,0_. Dashed lines are fits of the upper critical fields using a Ginzburg–Landau phenomenological model. The solid red line is a fit of the in-plane upper critical to the Werthamer–Helfand–Hohenberg expression. The Pauli limit is shown by the black dotted line. The inset shows the ratio of the in-plane and out-of-plane upper critical field as a function of temperature. (C) Angular dependence of the upper critical field *B*_c2_ for 1.65 K. For this panel, the critical field is defined as the field where the resistance has increased to 35% of the normal state resistance. The black and blue solid line are a fit with the 2D Tinkham formula  and the 3D anisotropic Ginzburg–Landau model , respectively. The inset shows the original data of angular-dependent magnetoresistance at 1.65 K. (D) The same as (B) but for a hole density of 1.10 × 10^13^cm^–2^. The inset displays the temperature dependence of the resistance for different parallel magnetic fields.

The origin of the enhanced *B*_*c2*__,∥_ requires further discussion. In traditional type-II superconductors, *B*_*c2*__,∥_ is governed by either the paramagnetic or orbital effect. Pair breaking induced by the orbital effect occurs at a field .^33^ Since a monolayer of 1T’-MoTe_2_ has an atomic thickness of only *d* ≈ 0.35 nm, *B*_*c2*,∥_^*orb*^(0K) is estimated to be as high as ∼58 ± 8 T for a density of 2.38 × 10^13^cm^–2^. An alternative way to estimate *B*_*c2*,∥_^*orb*^(0K) is the slope of *B*_c2,∥_(*T*) near *T*_*c*__,0_ ≈ 4.7 K. The data in Figure 3B yield  ≈ 20.7 *T*/K and then  ≈ 67 T. This field is far away from the experimentally determined *B*_c2,∥_. Hence, the orbital effect does not cause pair breaking here, as anticipated for 2D superconductors.

The paramagnetic effect originates from the preferential orientation of the spin in a field. The Zeeman energy difference for opposite spin electrons forming a Cooper pair can only be accommodated if it is smaller than the binding energy. This criterion yields the Pauli limit: .^25,26,38^ Here, Δ(0K) is the superconducting gap at 0 K, *g* is the Lande *g* factor, and *μ*_*B*_ is the Bohr magneton. Since *g* = 2 for BCS superconductivity, Δ(0K) Δ_*BCS*_ (0K) = 1.76*k*_*B*_*T*_c,0_ and the BCS Pauli limit becomes *B*_*p*_^*BCS*^ (0K) = 1.85*T*_*c*__,0_. An enhancement of the critical field above this limit implies either a larger Δ(0K) due to stronger pair interactions or an effective reduction of the Zeeman energy. The latter can be caused by strong spin orbit scattering or by a perpendicular spin orbit field that competes with the Zeeman field as is the case for Ising pairing.^35,38−41^

A fit of the *B*_c2_ data to the Werthamer–Helfand–Hohenberg model that considers the spin-paramagnetic effect has been added to Figure 3B. The model fits well when spin–orbit scattering is considered very weak: *λ*_*so*_ = 0.^38,39,42^ The fit yields a value of 5.3 for the Maki parameter α. With , we obtain *B*_*p*_(0K) ≈ 17.3 T. This corresponds to about twice the BCS Pauli limit *B*_*p*_^*BCS*^(0K) ≈ 8.7 T. A similar analysis has been performed in Figure 3D for a hole density of −1.10 × 10^13^ cm^–2^. In this case, the enhancement amounts to ∼2.2. In order to corroborate that this enhancement is not the result of strong spin orbit scattering but rather a strengthened pairing interaction, point-contact measurements were performed to extract the gap, a measure of the pairing strength. Figure 4A schematically shows the setup (method details in Section 12, SI). The point contact is a clean van der Waals-type contact formed by placing the monolayer on top of a Au electrode in a glovebox without any additional measures such as pressure.^43−45^Figure 4B displays several traces of the differential conductance *G*_*contact*_ = *I*_*ac*_/*V*_*ac*_ as a function of the dc voltage *V*_dc_ recorded on the Au/1T′-MoTe_2_ junction #1 of monolayer device D5 for an electron density of 1.38 × 10^13^cm^–2^. Temperature serves as an additional parameter. Figure 4C replots some data by normalizing to the 8 K data. Similar normalized data are shown for contact #2 in Figure 4E. Junction #2 was selected because it is in the metallic regime, whereas junction #1 is in the insulating regime.

**Figure 4:**
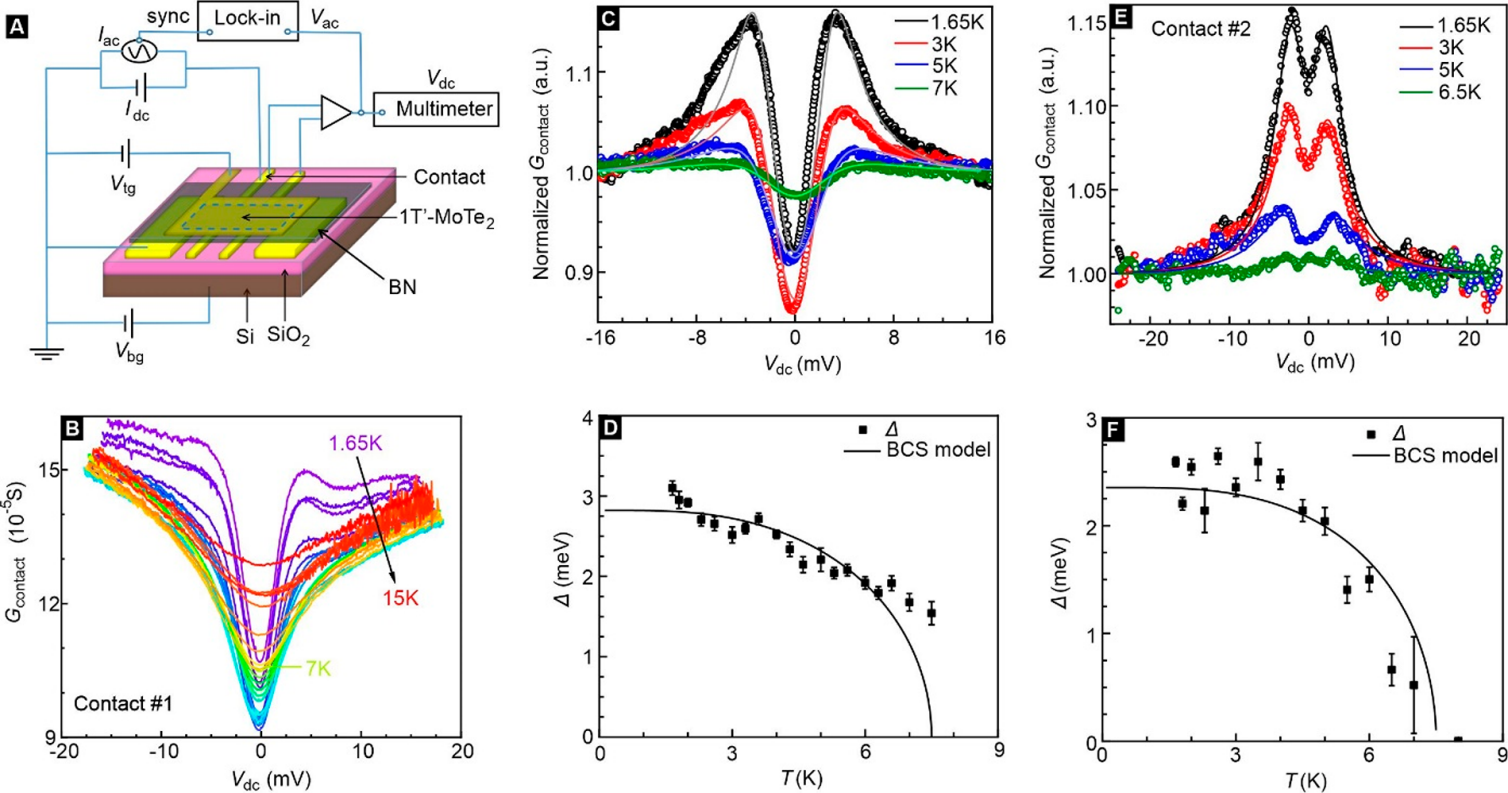
Point-contact spectroscopy in the superconducting regime. All data were taken on device D5 for a density of 1.38 × 10^13^cm^–2^ and for *B* = 0 T. (A) Schematic setup of the experimental arrangement used in the point-contact measurements. The applied ac bias current has a frequency of 17.77 Hz. The amplitude is set between 1 to 5 nA depending on the contact properties. (B) Temperature evolution of the differential conductance *G*_*contact*_ versus *V*_dc_ recorded on contact #1. (C) Theoretical analysis of the temperature-dependent normalized *G*_*contact*_ of contact #1 using the isotropic BTK model. (D) Temperature dependence of the gap amplitudes obtained from the BTK fit for contact #1. (E and F) Same as (C and D) but for contact #2.

We employ the extended single-band isotropic *s*-wave Blonder–Tinkham–Klapwijk (BTK) model to fit the point contact data^43−48^ in Figure 4D and 4F to obtain the temperature-dependent gap Δ(*T*). Included in these graphs is also the BCS model for the gap. The deviation of Δ(*T*) from the BCS relation in Figure 4D may be caused by spatial inhomogeneity.^49−51^ An estimate of Δ(0K) yields 2.8 ± 0.3 meV and 2.4 ± 0.3 meV for contacts #1 and #2. This allows us to determine gap ratios Δ(0K)/*k*_B_*T*_*c*__,onset_ of ∼4.3 and ∼3.7, respectively. These values are 2.1 to 2.5 times larger compared to the gap ratio of 1.76 for BCS weak-coupling. This matches well the enhancement of *B*_*c2*__,∥_ compared to the Pauli limit (2.0–2.2) and makes a convincing case that this is due to strengthened pairing interactions in 1T′-MoTe_2_ monolayers.^22,47^ Since the superconductor is in the dirty limit, spin–orbit scattering may play some role for *B*_*c2*__,∥_(0*K*),^10,40^ but it is likely only secondary (see Section 10, SI).

In Table 1 of the SI key superconductivity parameters measured on 1T′-MoTe_2_ monolayers are compared with other 2D superconductors. Superconductivity occurs at a density as low as ∼5 × 10^12^ cm^–2^, whereas in transition metal dichalcogenides exhibiting Ising superconductivity the required density is on the order of ∼10^14^ cm^–2^.^35,41,52,53^ Although the density required for superconductivity in monolayer 1T′-WTe_2_^9,10^ and twisted bilayer graphene^7,8^ is comparable, the onset of superconductivity occurs at a significantly larger temperature, *T*_*c*__,onset_ ≈ 7.5 K. The same holds for the upper critical field *B*_*c*__2_. It is also much larger than those in these other two systems. A large enhancement of *B*_*c2*__,∥_ has also been reported for other 2D superconductors exhibiting either type I or type II Ising pairing. Type I Ising pairing occurs in noncentrosymmetric superconductors with out-of-plane mirror symmetry due to a large out-of-plane spin orbit field such as for instance 2H-NbSe_2_,^35^ 2H-TaS_2_,^28^ and 2H-MoS_2_.^1,41^ Type II Ising pairing has appeared in few-layer stanene^52^ as well as PbTe_2_.^53^ Both are centrosymmetric but possess an unusual band structure near the Γ-point. Mating carriers reside in bands with different orbital indices, and bands are split due to spin orbit locking despite inversion symmetry. The symmetry and band structure properties of a 1T′-MoTe_2_ monolayer do not fit those of Ising type I or type II superconductors. Hence, the improved robustness of the superconductivity in 1T′-MoTe_2_ monolayers most likely results from enhanced pairing interactions.

## Supporting Information Available

Methods, sample thickness determination, carrier density estimates, electronic phase diagram, density and bias current dependence of the differential resistance, origin and estimate of the bulk gap, density dependence of the resistance at different temperatures and fields, low density regime, density dependence of the superconducting parameters, fit of the KLB Model to the *B*_c2,∥_ – *T*_c_ data, magnetic-field-induced superconductor-to-metal transition, point contact spectroscopy, monolayer device showing no evidence of a bulk gap, and comparison with other 2D superconductors (PDF)
