# Supplementary material for: Ambipolar Superconductivity with Strong Pairing Interaction in Monolayer 1T′-MoTe2
Source: Nano Lett. 2023 Aug 4;23(16):7516–23. doi: 10.1021/acs.nanolett.3c02033 (PMC10450800; doi:10.1021/acs.nanolett.3c02033)
Supplement: Supplementary file 1 — nl3c02033_si_001.pdf [file nl3c02033_si_001.pdf]

Supporting Information

Ambipolar superconductivity with strong pairing interaction in monolayer 1T'-$MoTe_2$

Fangdong Tang[1], Peipei Wang[2], Qixing Wang[1], Yuan Gan[2], Jian Lyu[2], Xinrun Mi[3], Mingquan He[3], Liyuan Zhang[2], Jurgen H. Smet[1]

[1] Max Planck Institute for Solid State Research, Stuttgart 70569, Germany.

[2] Department of Physics and Shenzhen Institute for Quantum Science and Engineering, Southern University of Science and Technology, Shenzhen 518055, China.

[3] Low Temperature Physics Laboratory, College of Physics & Center of Quantum Materials and Devices, Chongqing University, Chongqing 401331, China.

Content

1. Methods

2. Sample thickness determination

3. Carrier density estimates

4. Electronic phase diagram

5. Density and bias current dependence of the differential resistance

6. Origin and estimate of the bulk gap

7. Density dependence of the resistance at different temperatures and fields

8. Low density regime

9. Density dependence of the superconducting parameters

10. Fit of the KLB Model to the $B_{c2,\parallel} - T_c$ data

11. Magnetic-field-induced superconductor-to-metal transition

12. Point contact spectroscopy

13. Monolayer device showing no evidence of a bulk gap

14. Comparison with other 2D superconductors

15. References

1. Methods

Sample preparation. Bulk single crystals of 1T'-$MoTe_2$ were synthesized using the flux method with NaCl [1,2]. Monolayer and few layer films of 1T'-$MoTe_2$ were mechanically exfoliated from these bulk crystals using sticky tape inside a glovebox with a residual amount of water and oxygen of less than 0.1 ppm. Instead of transferring the flakes on the sticky tape directly onto the Si substrate, covered with the usual 300 nm thick dry thermal SiO_2 to analyze their thickness, a softer polydimethylsiloxane (PDMS) stamp was used as an intermediate to place the flakes on to such a Si substrate in order to obtain larger sized flakes. The flakes were typically not homogeneous in thickness, but were composed of the desirable monolayer or few layer region as well as an undesirable area with larger thickness. In order to remove the latter, a tear-and-release procedure using hBN on the PDMS stamp was used [3]. If the hBN was large enough to cover the monolayer or few layer region of the 1T'-$MoTe_2$ flake and only touched a small portion of the substrate itself, the part of the 1T'-$MoTe_2$ flake in touch with the hBN was torn off from the thicker part and picked up. This approach required no heating or other steps that would be detrimental for the sample quality. The hBN/1T'-$MoTe_2$ heterostructure was then transferred onto a substrate with pre-patterned Cr(5nm)/Au(20nm) contacts. Subsequently a graphite film was put on top of the hBN. It covers the whole area of the 1T'-$MoTe_2$ film and also touches a separate contact. This sequence of fabrication steps is illustrated with optical images in Fig. S1A-F.

To avoid any exposure to ambient air, packaging on a suitable chip carrier and bonding of the device with indium wires also proceeded inside the glove box. The heavily doped Si substrate served as a back-gate. A schematic of the sample is illustrated in Fig. 1A of the main text and the optical images of three completed devices (D2-D4) are shown in Fig. S1G-I. With the help of a load lock arrangement, the sample was mounted into the sample rod and subsequently transferred to the cryostat without exposure to ambient air. The entire procedure avoided not only air exposure of the 1T'-$MoTe_2$ sample, but also any exposure to solvents or elevated temperatures, all of which would cause a degradation of the sample quality. An accurate determination of the $MoTe_2$ layer thickness was performed a posteriori by combining three pieces of information: the optical contrast produced by the flake during exfoliation and stacking, the onset of the superconducting transition and confocal Raman spectroscopy after

completion of the magneto-transport measurements. The thickness determination works particularly well for monolayer and bilayer devices, as shown in Section 2, but becomes less reliable for thicker layers.

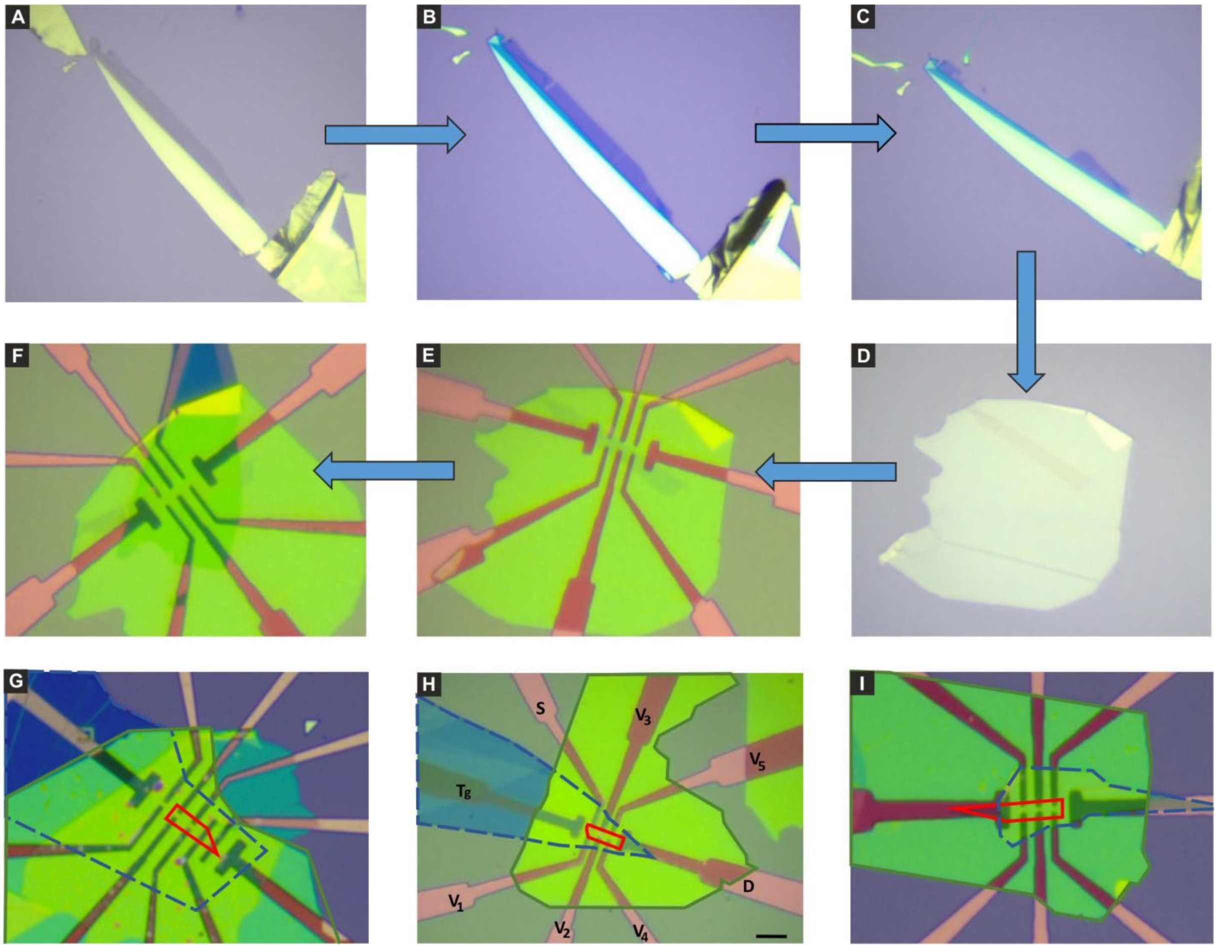

Fig. S1 | Optical images illustrating the sequence of device fabrication steps. Flakes of different thickness are produced by mechanical exfoliation using sticky tape. (**A**) The obtained flakes are transferred onto a PDMS stamp by pressing the tape with flakes onto the stamp. The region with weak contrast has monolayer thickness. It is about 30 µm long and is attached to 1T'-$MoTe_2$ of much larger thickness. (**B**), The flake on the PDMS stamp is transferred onto a doped Si substrate covered by a 300 nm thick thermal silicon oxide layer. A portion of the monolayer 1T'-$MoTe_2$ region is torn off from the thicker part by picking it up with an hBN film placed on top of the PDMS stamp. Panel **(C)** shows the remaining 1T'-$MoTe_2$ after tearing, while panel **(D)** is an image of the PDMS stamp with hBN as well as the monolayer 1T'-$MoTe_2$ piece. The hBN/$MoTe_2$ heterostructure was then transferred on top of another SiO_2/Si substrate on which a Cr(5nm)/Au(20nm) contact pattern was first deposited with the help of e-beam lithography and thermal evaporation (**E**). The flake touches the source and drain as well as the three contacts at the bottom. Finally, a graphitic layer is transferred on top of the device. It serves as the top gate **(F)**. Panels **(G)-(I)** show the optical images of three completed devices:

D2, D3 and D4. 1T'- $MoTe_2$, hBN and graphite are marked with a solid red, a solid green and a dashed blue line, respectively. The scale bar in panel **(H)** corresponds to 10 μm.

Measurements. The low-temperature magneto-transport measurements were performed in a dry cryostat system equipped with a superconducting magnet offering an axial field of up to 14 T and a variable temperature insert that allows temperature tuning between 1.6 K and 400 K. Low-frequency lock-in detection at 17.77 Hz was deployed for noise suppression to record longitudinal and transverse resistances. Measurement currents ranged between 1 nA and 10 μA depending on the two-point resistance of the sample. For both transport and point-contact measurements, the back-gate voltage, top-gate voltage and the dc bias current were applied with the help of a Keithley 2400 Source Measure Unit, a YOKOGAWA 7651 DC Source and an Agilent B2911A precision Source Measure Unit, respectively. The dc-voltage appearing as a result of the bias current was amplified by a voltage pre-amplifier and measured with an Agilent 34401A digital multimeter.

2. Sample thickness determination

In order to determine the thickness of the 1T'-$MoTe_2$ layer, we mainly use optical microscopy, Raman spectroscopy as well as the critical temperature of the superconducting transition. Optical images of layers with different thicknesses are illustrated in Fig. S2A. Layers with a thickness of up to three monolayers can be identified using the optical contrast. Raman spectra on flakes of different thicknesses are plotted in panel B. For layer thicknesses of less than four layers the position of the vibrational modes can be used to confirm the layer thickness [4]. These Raman spectroscopy results also help to "calibrate" the optical contrast needed for a certain layer thickness. The observed modes are denoted as P1 through P9 in Fig. S2B. In previous studies, the P1, P4, P5, P6, P8 and P9 modes were identified as A_g modes in the 1T' phase, whereas the P2, P3, and P7 modes were assigned as B_g modes. The position of the P1 mode displays the most obvious layer thickness dependence with the peak occurring at ~86 cm^{-1} for 1L, ~81 cm^{-1} for 2L, ~79 cm^{-1} for 3L and ~78 cm^{-1} for 4L. Beyond four layers the thickness determination becomes less reliable. The data in Fig. S2B show good agreement with previously published results [4].

The temperature-dependence of the resistance for samples of different layer thickness is summarized in Fig. S2C. The sheet resistivity increases significantly when the layer thickness decreases from four layers to one layer. At room temperature, the resistivity is about ~600 Ω

for devices with a thickness of 3 or 4 layers, ~2 kΩ for devices with two layers and ~5 kΩ for monolayer devices. For a thickness above two layers, the samples display metallic behavior and a critical temperature $T_{c,\mathrm{onset}}$ below 3 K. The $T_{c,\mathrm{onset}}$ increases significantly with layer thickness reduction as seen in the inset of Fig. S2C. This behavior is consistent with previously reported data in the literature [1,5]. For monolayer devices, a metal-to-insulator transition is observed at lower doping density, followed by a sharp resistance drop near 7-8 K signaling the superconducting transition. Hence, the $T_{c,\mathrm{onset}}$ can also serve as a convenient criterion to distinguish samples of different layer thickness.

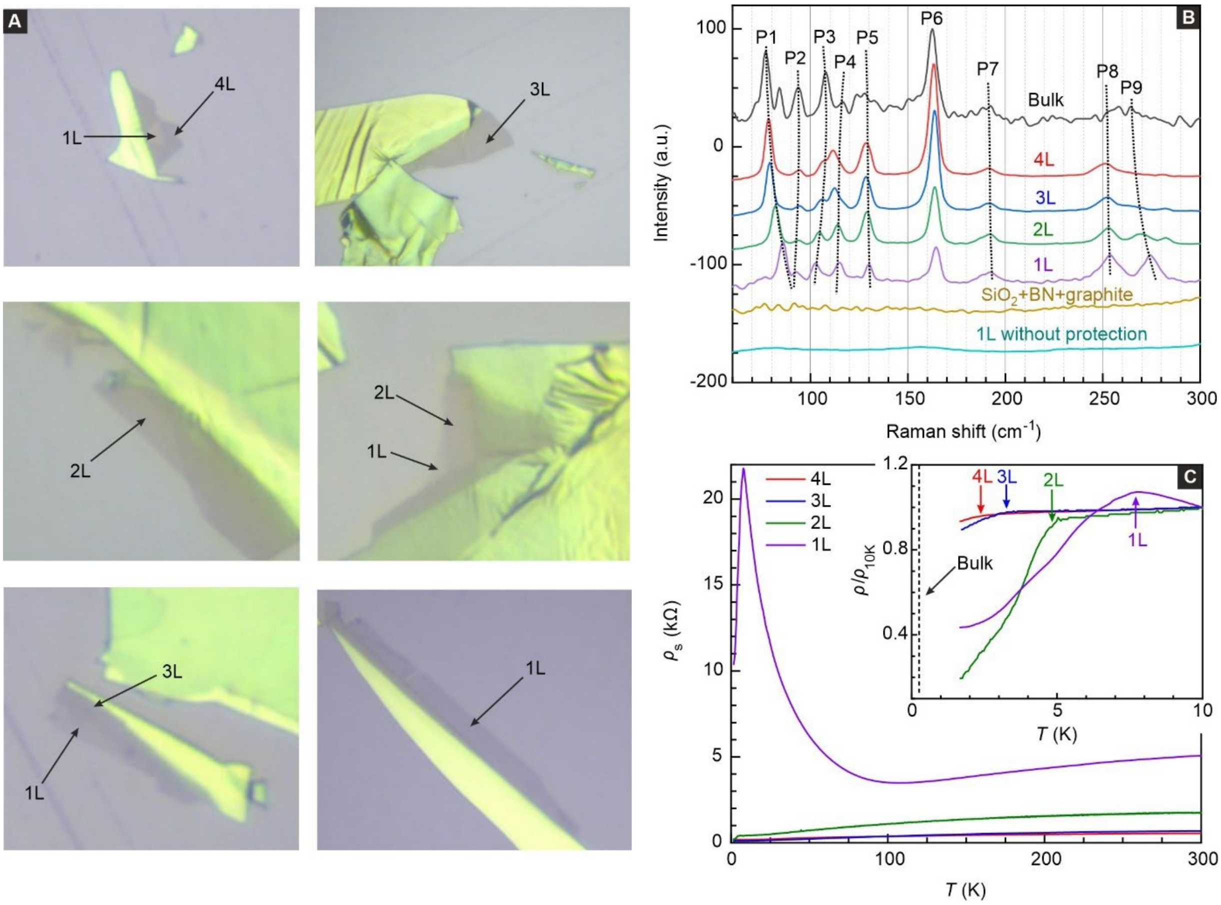

Fig. S2 | Different methods used to obtain information about the 1T'-$MoTe_2$ layer thickness. (A) Optical images of samples with a layer thickness between one and four layers. The optical contrast helps to identify the layer thickness. **(B)** Raman spectra on samples with different layer thickness from the monolayer to the bulk. The layer thickness or layer sequence of the samples used in this Raman study are marked for each trace. **(C)** Temperature-dependent resistivity of samples with 1T'-$MoTe_2$ layer thicknesses between one and four layers. The inset shows a zoom of each curve near the superconducting transition.

3. Carrier density estimates

The carrier density is estimated from a straightforward parallel plate capacitor model [6,7]

$$n_{\text{capacitance}} = \frac{c_{tg}V_{tg}+c_{bg}V_{bg}}{e} + n_0 = \frac{1}{e}\left(\frac{\varepsilon_0\varepsilon_r(\text{hBN})V_{tg}}{d_{tg}} + \frac{\varepsilon_0\varepsilon_r(\text{SiO}_2)V_{bg}}{d_{bg}}\right) + n_0.$$

Here e is the electron charge, n_0 is the density at zero back and front gate voltage induced by disorder, ε_0 is the permittivity of vacuum, $\varepsilon_r(\text{SiO}_2) \sim 3.9$ and $\varepsilon_r(\text{hBN}) \sim 4$ are the relative dielectric constants of SiO_2 and hBN, d_{bg}~300nm is the thickness of the SiO_2 thermal oxide covering the doped Si substrate, d_{tg} is the thickness of the hBN encapsulating layer, $c_{bg} = \frac{\varepsilon_0\varepsilon_r(\text{SiO}_2)}{d_{bg}}$ and $c_{tg} = \frac{\varepsilon_0\varepsilon_r(\text{hBN})}{d_{tg}}$ are the areal capacitances for the bottom and top gate, and V_{tg} and V_{bg} are the top and bottom gate voltages applied to the graphite layer and the heavily doped silicon substrate, respectively. Color renderings of the longitudinal resistance R_{xx} recorded across the parameter space spanned by the top and bottom gate voltages (V_{tg}-V_{bg}) at base temperature and zero magnetic field are shown in Fig. S3 for the monolayer devices D2 (panel A), D3 (panel B) and D4 (panel C).

The determination of the disorder induced density n_0 can in principle proceed in two different ways. In the first method it is assumed that the sample resistance reaches its maximum at zero average density. The resistance maxima should then correspond to $\frac{c_{tg}V_{tg}+c_{bg}V_{bg}}{e} + n_0 = 0$. Because the resistance maxima occur in the insulating regime, this procedure suffers from strong fluctuations in the data as is apparent in panel A-C of Fig. S3. Alternatively, it is possible to use carrier density data points extracted from Hall measurements in the high density regime and extrapolate n_0 from a fit of the data to the expression $n_{\text{Hall}} = \frac{c_{tg}V_{tg}+c_{bg}V_{bg}}{e} + n_0$. This method too is not without difficulties. Fig. S3D displays Hall resistance traces recorded at different pairs of the back and top gate voltages. The behavior of the Hall resistance becomes anomalous at lower density when entering the insulating regime. We would expect the Hall resistance to rise with decreasing density, yet the Hall resistance drops. This suggests that in this regime the net average density is low, but there are co-existing hole and electron populations which prevent the proper extraction of the charge carrier density from the Hall resistance formula assuming a single charge carrier type. In Fig. S3E the experimentally determined Hall densities ignoring this issue are compared with the densities determined from the capacitor model. The Hall density deviates significantly from the expected linear capacitor model behavior for densities below 1.5×10^{13} cm^{-2}. This effectively constrains the useable density range for the extraction of n_0 and limits its accuracy. Since the longitudinal resistance

increases substantially even though the drop in the Hall resistance suggests the availability of both an electron and hole population, there is apparently more to it. The insulating state may be a correlated insulator or may host edge states within the gap, as mentioned in previous publications [5,7]. Due to the requirement of avoiding exposure of the $MoTe_2$ to air, it is not possible to etch the sample in a regular Hall bar geometry with well-defined contact terminals. The contact area and sample area are not well separated. All of these issues complicate the interpretation of the observed Hall resistance behavior. While both methods for extracting n_0 are prone to uncertainty, this has no impact on the main results. Here we list some obtained values for n_0 using either one of both methods. The details of the substrate are well known. For a 300 nm dry thermal SiO_2, $\frac{c_{bg}}{e}$ equals 7.19×10^{10} cm^{-2} for all devices. For device D2 and D3, the thicknesses of the top hBN is measured to be equal to 42 nm and 40 nm using atomic force microscopy (AFM) resulting in a $\frac{c_{tg}}{e}$ of 5.3×10^{11} cm^{-2} and 5.5×10^{11} cm^{-2}, respectively. Using the first method, the intercept of the linear fit to the resistance peaks in panel A and B then yields $n_0 = 9.3\times10^{12}$ cm^{-2} for device D2 and $n_0 = 3.4\times10^{12}$ cm^{-2} for device D3. For device D4 gate dependent Hall measurements were used instead (Fig. S3E). For this device, we get $\frac{c_{tg}}{e} = 7.1\times10^{11}$ cm^{-2} and $n_0 = 5.7\times10^{12}$ cm^{-2}. For device D5, $\frac{c_{tg}}{e}$ equals 8.3×10^{11} cm^{-2}, while we obtain a disorder induced density n_0 of 1.2×10^{12} cm^{-2} using a fit to the resistance maxima (first method).

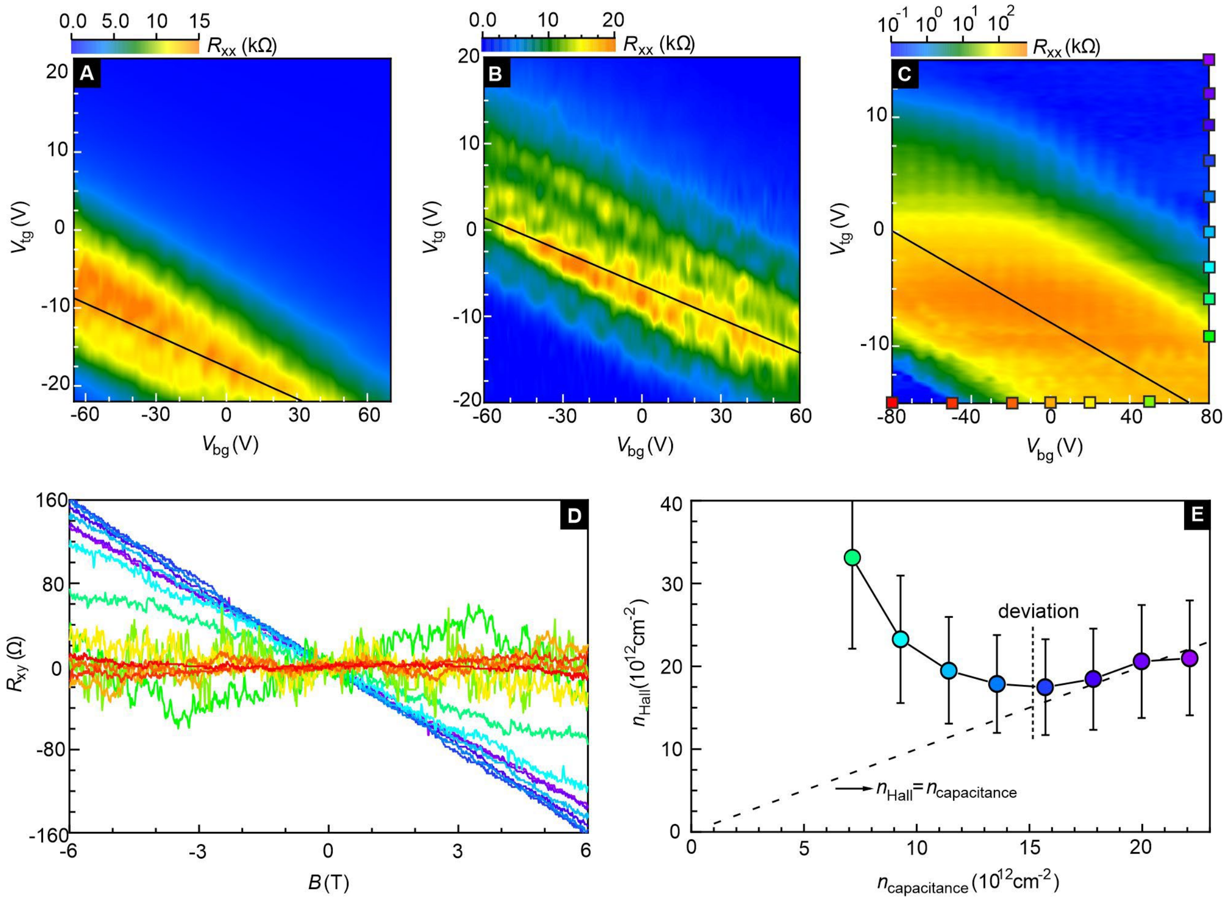

Fig. S3 | Carrier densities extracted from the capacitance model and from Hall measurements. (A-C) Gate dependence of the longitudinal resistance R_{xx} in the V_{tg}-V_{bg} plane for device D2, D3 and D4 from left to right. Solid black lines mark the estimated position of charge neutrality, i.e. average zero carrier density. **(D)** Hall measurements recorded on device D4 at the gate voltages marked in panel **C** using squares of the same color as each of the Hall resistance traces. **(E)** Comparison of the carrier density extracted from the Hall resistance (n_{Hall}) and the carrier density calculated from the capacitor model ($n_{\mathrm{capacitance}}$). The coloring of the experimental Hall density data points again corresponds to the gate voltage pairs marked with a square of the same color in panel C.

4. Electronic phase diagram

All monolayer devices exhibit the same phase transitions from an insulating state at low density to a superconducting state for larger electron or hole doping. Fig. S4A-C illustrate the evolution of the RT curves below 15 K for different electron and hole densities. The resistance in the insulating regime is the highest for device D4 suggesting fewer residual charge carriers at zero average density, whereas the low resistance in device D2 in the insulating regime indicates a higher degree of inhomogeneous disorder. Panels D-F display color renditions of the resistance in the temperature versus density plane for device D2, D3 and D4. In each case, the parameter

space can be subdivided in areas where the samples exhibit normal metallic behavior (NM), insulating behavior and superconducting behavior (SC). Fig. S5 shows the evolution of the *RT* curves for different densities as in Fig. S4, but for a much wider temperature range (1.6 to 200 K). The appearance of a metal-insulator transition happens in all devices at temperatures below about ~100 K as marked with a dashed black line. This heralds the opening of a bulk gap in the monolayer.

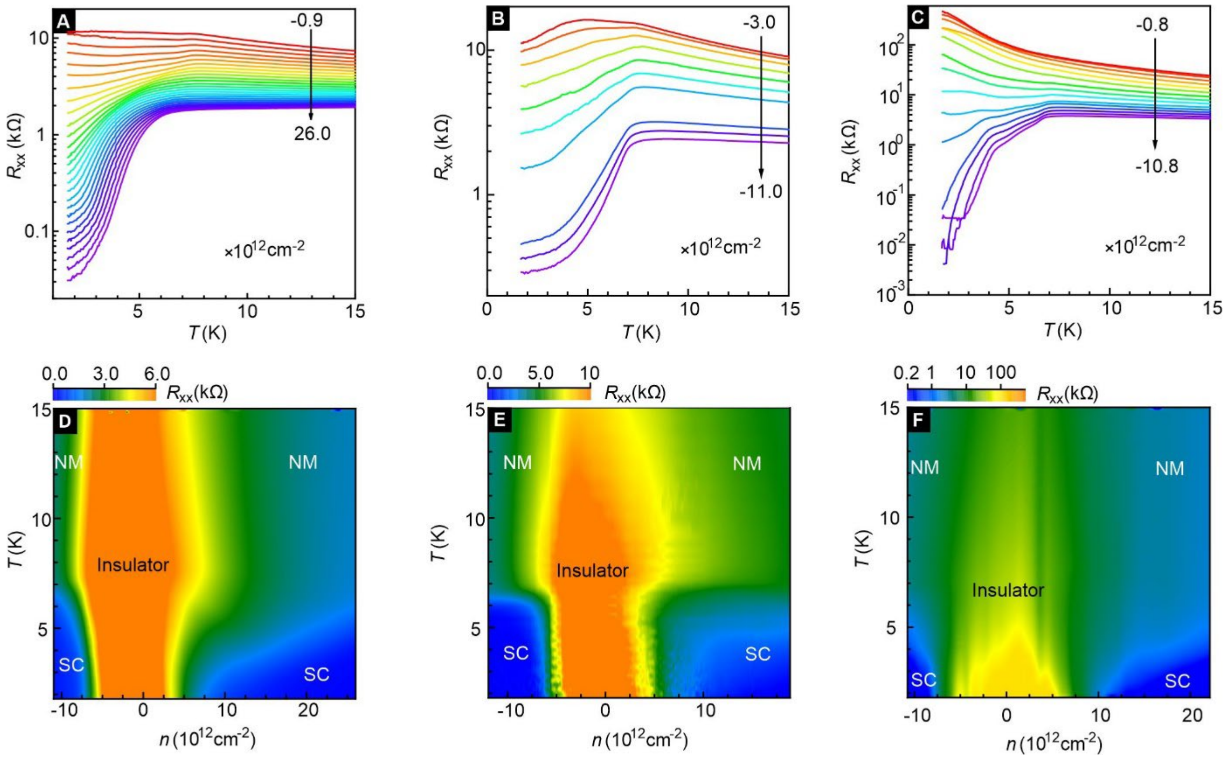

Fig. S4 | Density dependent *RT* curves and resistance color maps as a function of density and temperature up to 15 K. (A and B) Temperature-dependent resistance for different electron and hole carrier densities in device D2, respectively. **(C)** The same as (**A**) but for device D4 for different hole densities. (**D-F)** 2D color maps of the resistance in the plane spanned by temperature and density for device D2, D3 and D4. The different phases are marked in each diagram (insulator, normal metal - NM, superconductor - SC).

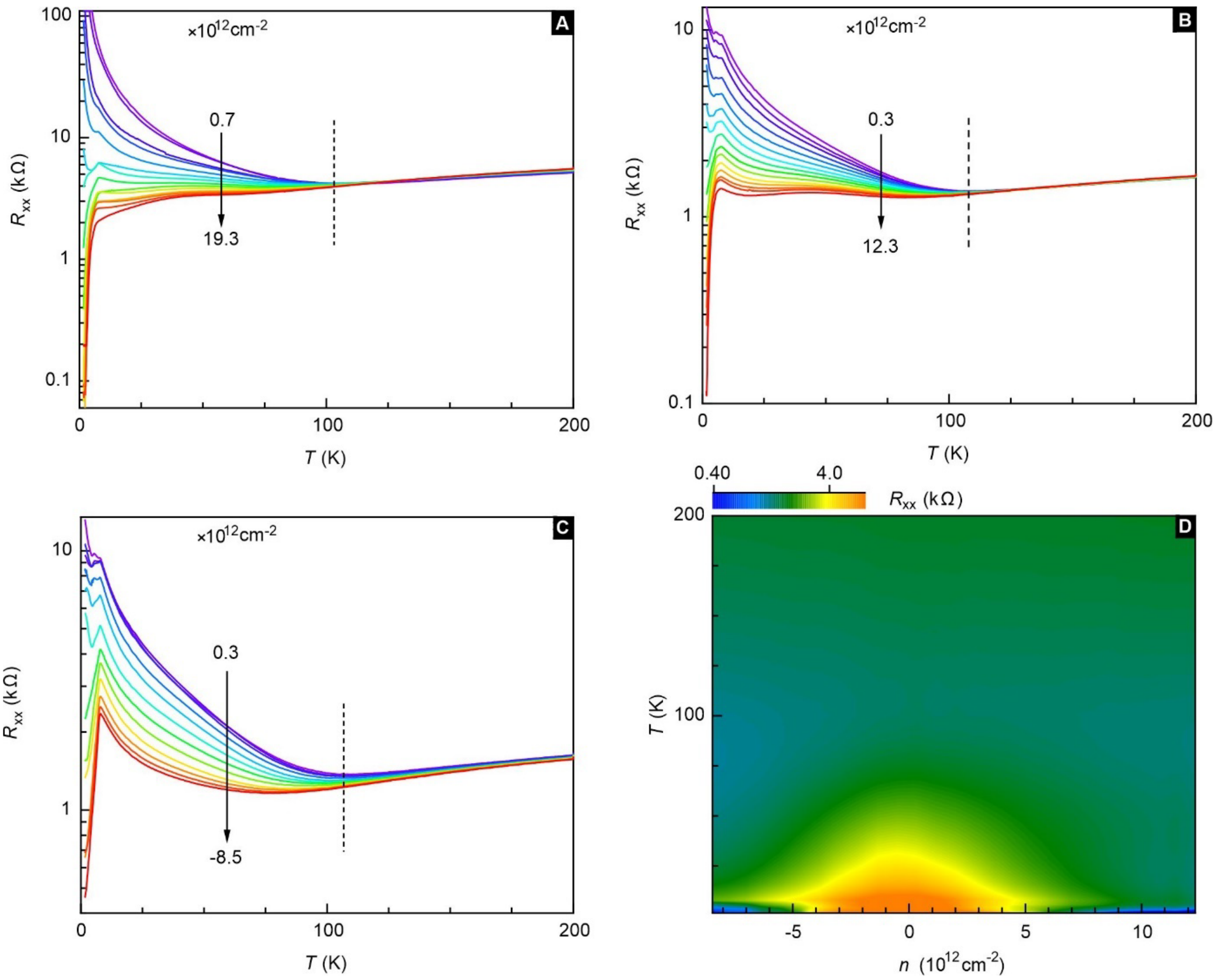

Fig. S5 | Density dependent *RT* curves and resistance plot as a function of density and temperature up to 200 K. (A) Temperature-dependent resistance traces for different electron densities recorded on device D4. The dashed black line marks the temperature at which a metal-to-insulator transition appears when tuning the density. (**B and C)** Same as (**A**) but for device D5 for different electron (**B**) and hole (**C**) densities. **(D)** 2D color map of the resistance in the parameter plane spanned by the density and temperature for device D5.

5. Density and bias current dependence of the differential resistance

Fig. S6 displays the behavior of the differential resistance dV/dI as a function of the bias current and the carrier density for three different monolayer samples: D2, D3 and D4. The top panels are waterfall plots whereas the bottom panels are color renderings of the differential resistance in the dc bias current and carrier density plane.

The large dV/dI peaks for zero bias current reflect the insulating state (Ins) at low densities. The peak value strongly depends on the device, which we attribute to the significant variation in the residual spatially inhomogeneous carrier density. As the electron (e) or hole (h) density is raised, the peak in the differential resistance at zero bias current vanishes and the differential

resistance drops to zero at low bias current instead. This region of zero differential resistance expands with increasing electron or hole density and the critical current where the differential resistance becomes non-zero moves to higher bias current. The density dependence of the critical current is not symmetric when comparing electron and hole doping. This presumably reflects the lack of symmetry in the band structure.

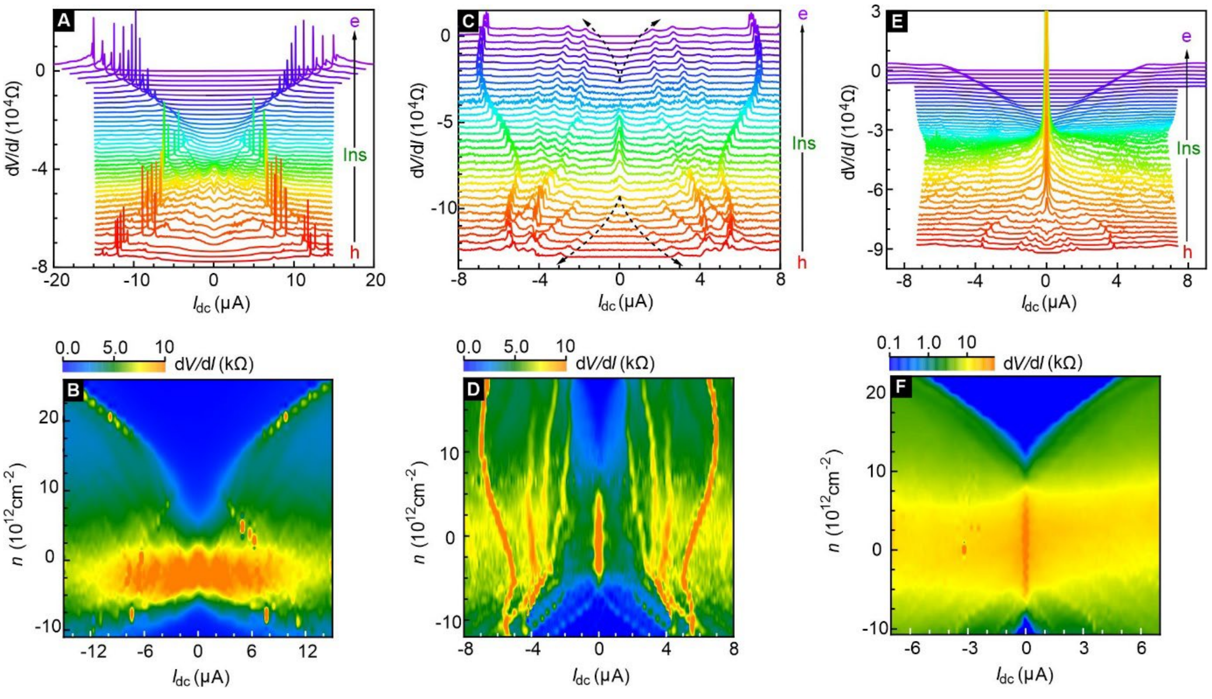

Fig. S6 | Differential resistance dV/dI as a function of dc bias current and charge carrier density. (A) Waterfall plot of the differential resistance recorded on device D2 at T = 1.7 K and B = 0 T for densities ranging from -1.10×10^{13} cm^{-2} to 2.60×10^{13} cm^{-2}. Each curve is offset by 2 kΩ. **(B)** 2D color plot of the differential resistance data shown in panel **(A)**. (**C** and **D)** The same as (**A)** and (**B**) but for device D3 with densities ranging from -1.20×10^{13} cm^{-2} to 1.88×10^{13} cm^{-2}. The offset in (**C**) is 4 kΩ. (**E** and **F)** The same as (**A)** and (**B**) but for device D4 with densities ranging from -1.08×10^{13} cm^{-2} to 2.21×10^{13} cm^{-2}. Each curve is offset by 2 kΩ in (**E**).

In some samples spikes appear in the dV/dI further away from the zero bias current point. A very clean example is seen in Fig.2C of the main text. This data set is reproduced here in Fig. S6C-D. These spikes can be traced across the entire scanned density range. To identify unequivocally the origin of these spikes is difficult and beyond the scope of the present work as this requires local probe methods. However, a scenario where isolated superconducting domains, that exist within the sample, suddenly turn normal at sufficiently high bias current, is compatible with the available experimental observations. The shape of the features would as

in our experiment depend on the details of the disorder landscape, which determines how such superconducting domains expand or shrink as the average density is tuned. The fact that in some other samples such features do not appear or are less pronounced as in Fig. S6A-B corroborates the importance of the disorder details.

6. Origin and estimate of the bulk gap

The opening of a band gap in monolayer 1T'-$MoTe_2$ has been controversially debated in the literature. Different DFT calculations and experimental measurements yield significantly different results whether a gap exists or not and how large it is, if a gap exists. The authors of Ref. 8 compiled a valuable table (Table I in this reference) [8]. It unveils large discrepancies between different studies and approaches. Whether spin-orbit coupling (SOC) in 1T'-$MoTe_2$ is strong enough to open a gap remains unclear. Compared with 1T'-WTe_2, the SOC in 1T'-$MoTe_2$ is much weaker. However, the onsite Coulomb repulsion of the Mo d-electrons is stronger than for W and this strong electron-electron (e-e) interaction must be considered as it can influence the opening of a band gap. Recent progress on 1T'-WTe_2 has suggested that also exciton condensation may play an important role for the gap physics [9,10]. Hence, three different mechanisms have been identified that can contribute to the formation of a gap: spin-orbit coupling, electron-electron interaction and an excitonic instability. This richness complicates the assessment of whether and how strong a gap should form and what the predominant origin of such a gap would be. Our experiments are not able to identify the main cause of the gap. Point is made very clear and any statements about the origin of the gap are softened accordingly. However, we consider the existence of a gap in our monolayer devices of 1T'-$MoTe_2$ beyond doubt. Some of us previously reported a magnetotransport study on multilayer 1T'-$MoTe_2$ devices [11]. At that time, devices were not fabricated such that they are not exposed to ambient conditions or elevated temperatures during processing and sample mounting. While a minor degree of oxidation might just cause some extra doping, more oxidation will induce significant disorder and the majority of the multilayer devices showed insulating behavior due to this disorder. As a matter of fact, it has been a common observation that samples turn insulating after oxidation in earlier studies on various 2D materials. For instance in Ref. 2 and 11 insulating behavior was observed in thicker 1T'-$MoTe_2$ devices. If the samples are protected well, multilayer 1T'-$MoTe_2$ devices become metallic and superconducting instead, as evident from the work here as well as previous works [1,5]. A clear distinction must be made between

insulating behavior as a result of such oxidation induced extrinsic disorder and insulating behavior caused by the presence of a gap induced by any of the above three intrinsic mechanisms. We think it is fair to argue that here we are dealing with the second case. After all, multilayer samples (1T'-$MoTe_2$ as well as 1T'-WTe_2) no longer show insulating behavior, but systematically exhibit metallic behavior because the fabrication technology efficiently prevents oxidation. If oxidation would remain relevant, this would not be the case. The most plausible explanation for monolayer samples to exhibit insulating behavior is the appearance of a gap.

It is possible to estimate the size of the bulk gap Δ by performing an Arrhenius plot analysis of the temperature dependent resistance data in the regime where the sample exhibits insulating behavior: $R_{xx}(T)\sim \exp\left(\frac{\Delta}{2k_BT}\right)$ [12]. The analysis should be performed at gate bias points where the chemical potential is located inside the bulk gap and the average density is low. Inhomogeneous disorder, sufficiently large to generate a landscape of electron and hole puddles, will likely cause an underestimation of the gap size. Hence, higher quality samples with less disorder are more suitable for such an analysis. In Fig. S7A an Arrhenius fit is performed on the resistance data obtained on device D4 with the highest quality at a density of 7×10^{11} cm^{-2}. The main graph plots the temperature dependent resistance on a logarithmic scale, whereas the inset displays the Arrhenius fit (solid red line) to the conductance:

$$\frac{1}{R_{xx}} = G_{xx}(T)\sim \exp(-\frac{\Delta}{2k_BT}),$$

focusing on the high-temperature regime between 20 and 100 K. This yields a value for the bulk gap of ~7.3meV. The Arrhenius fit deviates significantly at temperatures below 20 K. This temperature regime is described well by the expression for variable-range hopping (VRH) in a 2D system with localization [$G_{xx}(T)\sim \exp\left(-(\frac{T_0}{T})^{\frac{1}{3}}\right)$], as seen in the inset of Fig. S7A (solid black line). Even if so, other potential mechanisms can't be totally ruled out [13]. In another device D5, the estimated bulk gap is ~7.0 meV for a net density of 3×10^{11} cm^{-2} (Fig. S7B). This is close to the gap value obtained on device D4 in panel A.

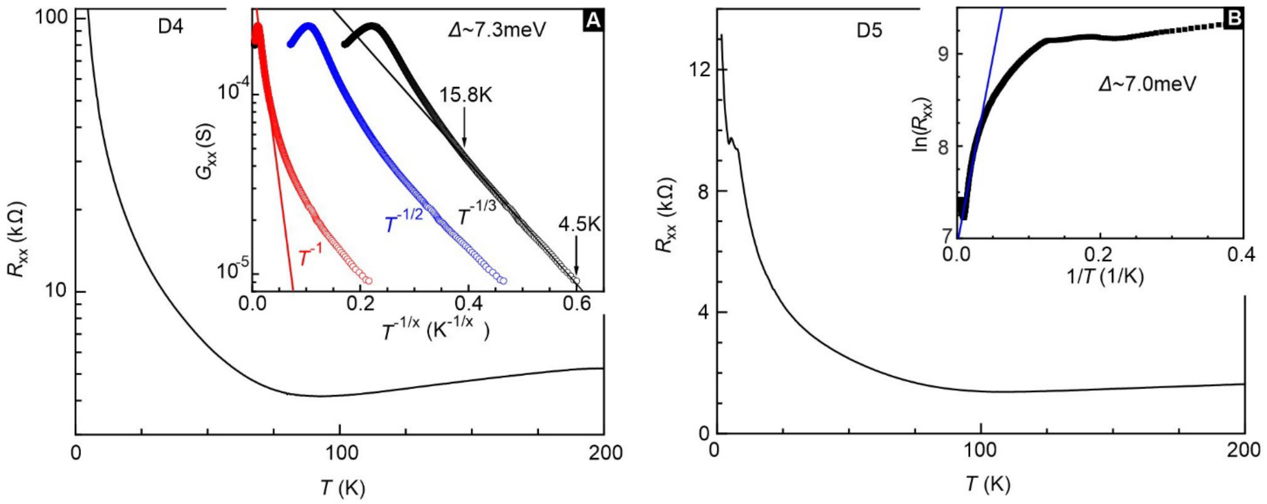

Fig. S7 | Estimate of the bulk gap for samples with different degrees of inhomogeneous disorder. (A) Temperature-dependent resistance for device D4 in the insulating regime at a density of 7×10^{11} cm^{-2}. The inset shows an Arrhenius fit for temperatures from 20 to 100 K (red line) and a fit assuming variable range hopping in the temperature interval from 4 to 20 K (black line with $T^{-1/3}$). The blue curve is shown for the sake of comparison with a $T^{-1/2}$ dependence. (**B)** The same as **(A)** but for device D5 at a net density of 3×10^{11} cm^{-2}.

7. Density dependence of the resistance at different temperatures and fields

Fig. S8 plots the density dependence of the resistance for different temperatures and magnetic fields for devices D2, D4 and D5. By sweeping the gate voltage, the chemical potential is tuned continuously from the valence band into the conduction band. This results in ambipolar transport behavior with insulating behavior in between when the average density is small. The highest resistance in the insulating regime is strongly sample-dependent. It ranges from ~10^4 ohms to ~10^5 ohms, as seen in panels A, C and E of Fig. S8. There is no clear evidence for the existence of topological edge states. Such edge states would cause a resistance peak with a quantized value of about $h/2e^2$. Resistances are much larger. Moreover, they are sample-dependent [14,15]. Panels B, D and F plot the magnetic field dependence of the resistance for samples D2, D4 and D5. The resistance in the insulating regime increases gradually with magnetic field. This contradicts the expected behavior for quantum spin Hall edge states when time-reversal symmetry gets broken in the presence of a small magnetic field. This lack of experimental signatures for edge states may be the result of remaining disorder and the inevitably associated scattering. Short-channel devices are more appropriate to mitigate at least to some extent the influence of disorder for edge state related transport [16,17].

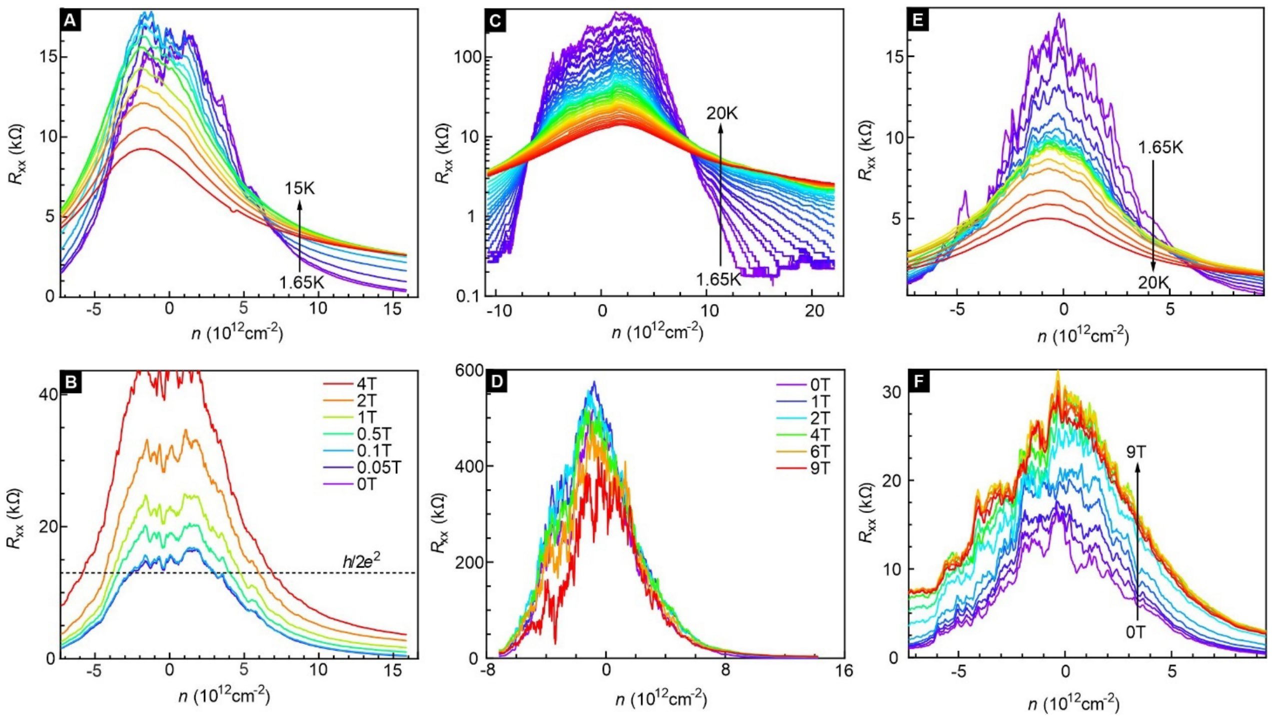

Fig. S8 | Density dependence of the resistance at different temperatures and magnetic fields. (A) Density dependent longitudinal resistance data for device D2 in the absence of a magnetic field and with temperature as a parameter. The temperature range is 1.65 to 15 K in steps of about 1 K. (**B**) Same as in (**A**) but at a fixed temperature of 1.65 K and with the magnetic field as the parameter. (**C** and **D**) Same as (**A**) and (**B**) but for device D4. The covered temperature range is from 1.65 to 20 K in approximately 0.25 K steps. (**E** and **F**) Same as (**A**) and (**B**) but for device D5. The temperature range from 1.65 to 20 K is covered in 1 K steps (**E**). The magnetic field varies from 0 to 9 T in 1T steps.

8. The low density regime

In the low density regime, the resistance behaves as an insulator for temperatures typically down to about 7-8 K marked by the gray area in the exemplary density-dependent RT traces recorded on device D1, D2 and D4 in Fig. S9A-C. The kink in the traces in this temperature regime signals the onset of the superconducting transition and does not change significantly when varying the doping level. This is reminiscent of a superconductor-to-insulator transition in granular films of various metals [18,19]. The behavior is attributed to the existence of isolated superconducting puddles in an insulating background [20], as shown in a cartoon like fashion in the inset of Fig. S9C. This landscape originates from the spatial density inhomogeneity across the sample where areas of high-density remain conducting. Upon reducing the temperature below the transition temperature $T_{c,\mathrm{onset}}$ Cooper pairs form within these conducting puddles and cause at least a kink or a pronounced downturn of the resistance. The carrier density

averaged across the entire sample area is still very small, but inside these puddles the carrier density should be on the order of ~10^{12} cm^{-2} and can be tuned by changing the applied gate voltages. The transition temperature $T_{c,\mathrm{onset}}$ does not or depends only weakly on the applied gate voltage even down to lowest average sample densities as can be seen in Fig. S9D. The traces plotted in panel A and B for higher average carrier densities indicate that the resistance continues to decrease as temperature is lowered, but the resistance values remain non-zero and large. This suggests that there is only weak link coherence among the puddles and overall superconductivity cannot be achieved.

The existence of superconducting puddles is also corroborated in temperature dependent resistance data in the presence of a perpendicular magnetic field. When the magnetic field is increased, the superconducting puddles should gradually convert into the normal state at relatively high fields (> 2T) and the insulating behavior should then persist also for temperatures below 7 K. This is indeed observed in perpendicular field data plotted in Fig. S10. The low-temperature resistance reaches values as high as ~10^5 ohms when the superconducting puddles are entirely quenched in high magnetic fields.

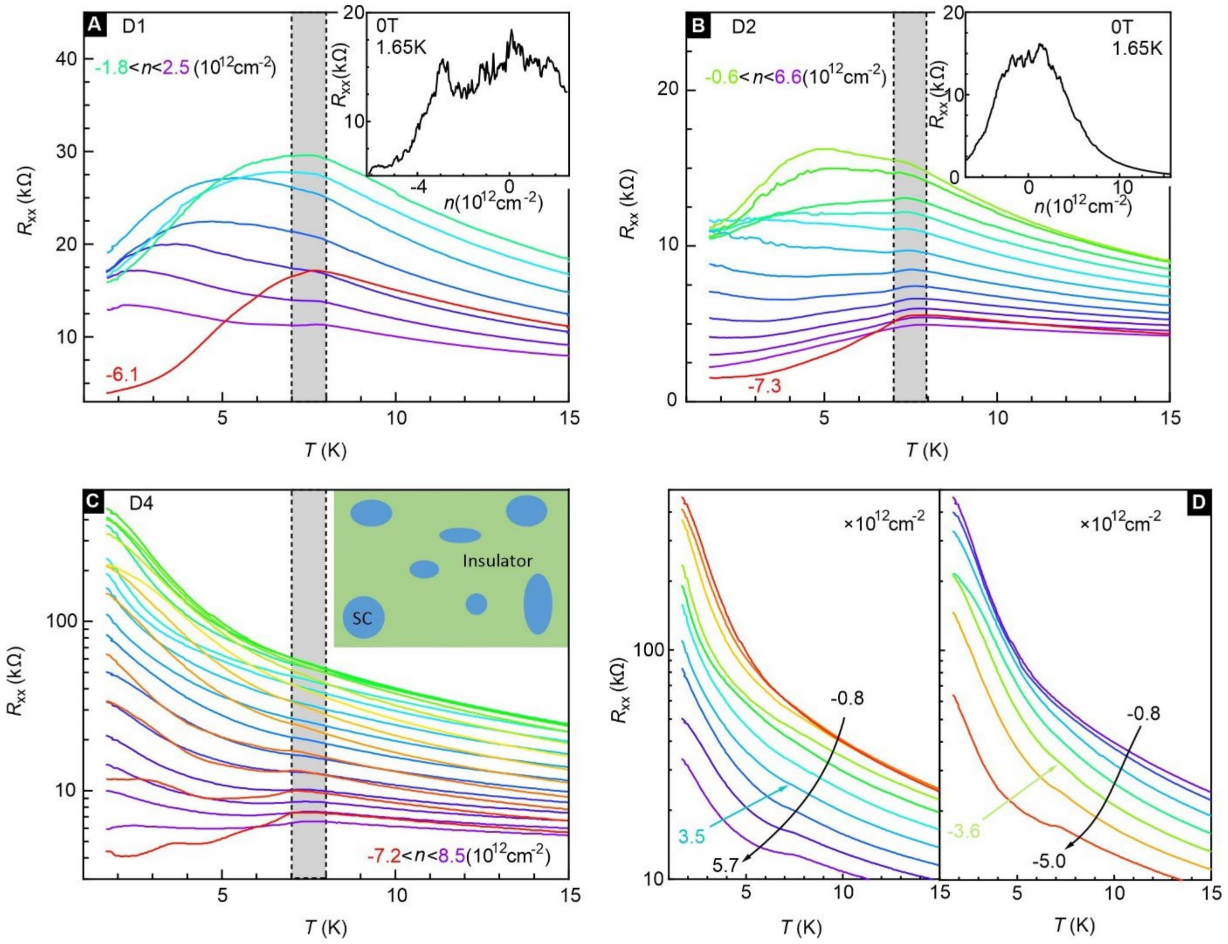

Fig. S9 | Superconducting puddles in the insulating regime. (A) *RT* curves recorded on device D1 for varying carrier densities (red curve: -6.1×10^{12} cm^{-2}, green to purple curves: $-1.8<n<2.5\times10^{12}$ cm^{-2}). The gray area marks the common transition-temperature region. The inset shows the gate-dependent resistance at 1.65 K and zero magnetic field. **(B)** Same as (**A**) but for device D2 (red curve: -7.3×10^{12} cm^{-2}, green to purple curves: $-0.6<n<5.3\times10^{12}$ cm^{-2}. **(C)** Same as (**A**) but for device D4 ($-7.2<n<8.5\times10^{12}$ cm^{-2}). The inset displays the schematic of the superconducting puddles. **(D)** Same as **(C)** but divided into two panels for electron (left panel) and hole (right panel) doping, respectively.

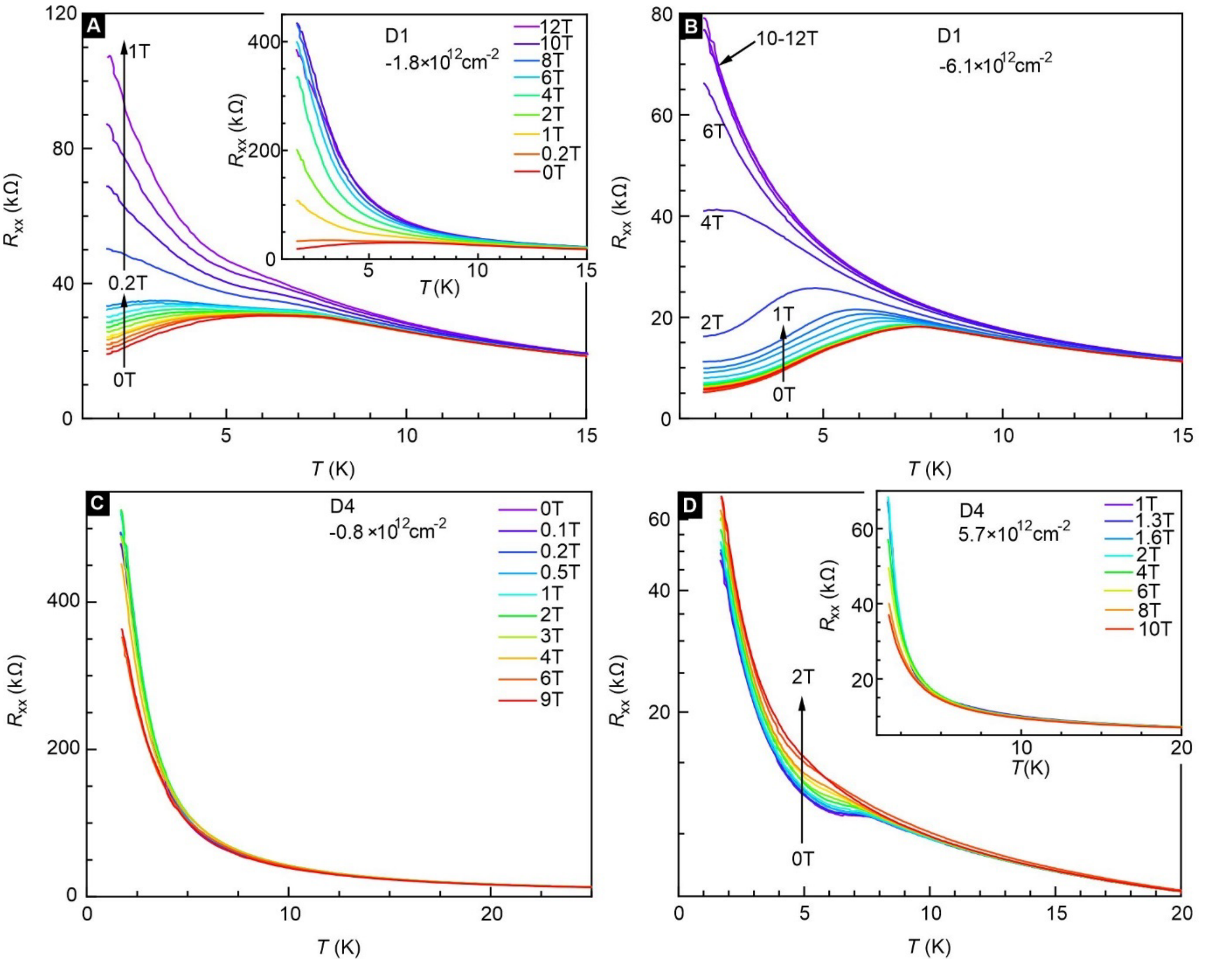

Fig. S10 | Magnetic field-dependent behavior in the insulating regime. (A) *RT* curves recorded in the presence of different perpendicular magnetic fields (from 0 to 1 T) on device D1 for an average hole density of -1.8×10^{12} cm^{-2}. The inset displays data recorded at higher magnetic fields (0-12 T). **(B)** Same as **(A)** but for a hole density of -6.1×10^{12} cm^{-2}. **(C)** Same as **(A)** but for device D4 for a hole density of -8×10^{11} cm^{-2}. **(D)** The same as **(C)** but for an electron density of 5.7×10^{12} cm^{-2}.

9. Density dependence of the superconducting parameters

In Fig. S11, we summarize the density dependence of some key sample and superconductivity parameters, such as the superconducting transition temperature $T_{c,0}$, the out-of-plane critical field $B_{c2,\perp}$, the BCS coherence length ξ_0 and the mean free path l_{mfp}. Within the density range we can cover, the superconductivity strengthens with density, since both $T_{c,0}$ in Fig. S11A and $B_{c2,\perp}$ in Fig. S11B continue to increase with increasing density without observing a maximum that would signal optimal doping. Note that a distinction must be made between the superconducting transition temperature $T_{c,0}$ determined from the criterion that the resistance drops to 50% of the normal state resistance and the temperature $T_{c,onset}$ determined from a kink or downturn of the resistance marking the onset of the superconducting transition. A resistance value of 50% of the normal state resistance reflects a macroscopic average across the entire sample and hence $T_{c,0}$ is a "global" property. $T_{c,0}$ therefore diminishes upon reducing the carrier density as seen in Fig. S11A. This behavior is expected. In contrast, $T_{c,onset}$ does not reflect global behavior, but local behavior and at zero magnetic field varies little even in the low-density regime. We attribute this to the spatial density inhomogeneity across the sample. Even in the insulating regime, some areas exist where the local carrier density is sufficiently high to enter the superconducting regime. Hence, superconducting puddles are present when the temperature approaches $T_{c,onset}$ even in the insulating regime. When the temperature drops below $T_{c,onset}$, Cooper pairs form in these areas and cause at least a kink or downturn in the resistance. $T_{c,onset}$ therefore does not vary with the average carrier density as $T_{c,0}$ does.

In order to judge whether the superconductor is in the dirty limit ($\xi_0 \gg l_{mfp}$) or the clean limit ($l_{mfp} \gg \xi_0$), the BCS coherence length is compared with the mean free path for different densities [21,22]. The mean free path follows from the Drude model: $l_{mfp} = h/(e^2\rho_s\sqrt{g_s g_v \pi n_e})$ with ρ_s the sheet resistivity, n_e the net charge carrier density. For electron densities, $g_s = g_v = 2$ to account for both the spin and valley degeneracy. For hole doping, $g_s = 2$ and $g_v = 1$ [5,6]. We can obtain the BCS coherence length from the in-plane Ginzburg-Landau (GL) coherence length using the relation $\xi_0 = 1.35\,\xi_{GL}(0\text{K})$. The in-plane coherence length $\xi_{GL}(0\text{K})$ is calculated using the expression $B_{c2,\perp}(0\text{K}) = \frac{\phi_0}{2\pi\xi_{GL}^2}$, where ϕ_0 stands for the superconducting flux quantum $h/2e$ and $B_{c2,\perp}(0\text{K})$ is the out-plane critical field at 0 K. The latter is obtained from a linear extrapolation of temperature dependent measurements of the out-of-plane critical field. A comparison of l_{mfp} and ξ_0 is displayed in Fig. S11C for device D4 and in Fig. S11D for device D2. Since ξ_0 is always larger than l_{mfp}, both devices are in the dirty limit. When decreasing the carrier density down to the insulating regime, ξ_0 increases,

whereas l_{mfp} decreases. Hence, the devices are located even deeper into the dirty regime with decreasing density.

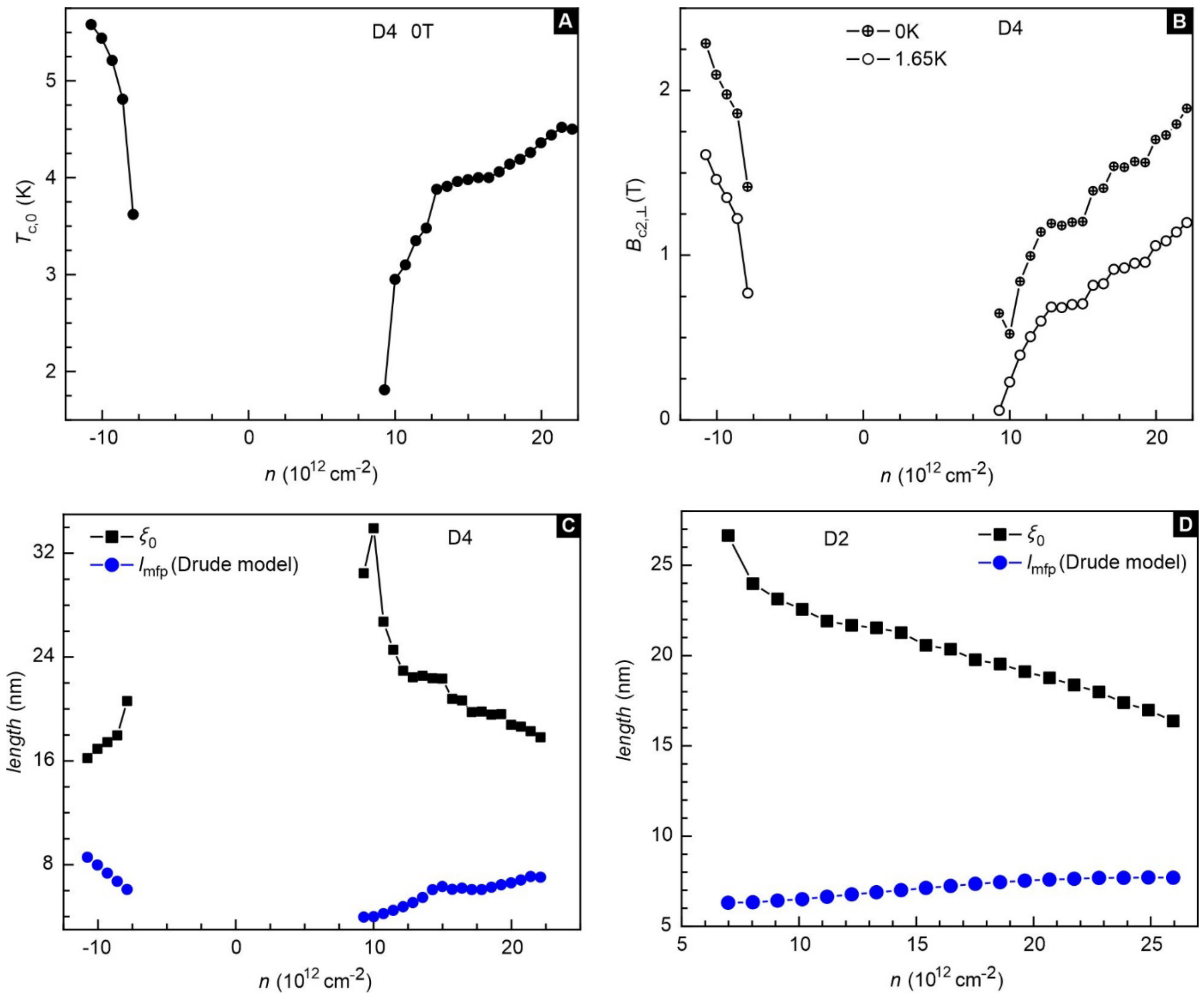

Fig. S11 | Density dependence of the superconducting parameters. Density dependence of $T_{c,0}$ (**A**) and $B_{c2,\perp}$ at 1.65 K and 0K (**B**) for device D4. $B_{c2,\perp}$(0K) is obtained from a linear extrapolation of temperature dependent $B_{c2,\perp}$ measurements. **(C)** Comparison of the estimated in-plane BCS coherence length ξ_0 (black symbols) and mean free path l_{mfp} (blue symbols) for device D4. **(D)** The same as **(C)** but for device D2**.**

10. Fit of the KLB model to the $B_{c2,\parallel} - T_c$ data

Since the superconductivity in our samples occurs in the dirty regime, spin-orbit scattering can be a potential mechanism enhancing the in-plane critical field $B_{c2,\parallel}$ with respect to the BCS

Pauli limit. This is captured by the KLB theory. It yields the following expression for the in-plane field dependence of the critical temperature [6,23]

$$\ln\left(\frac{T_c}{T_{c,0}}\right) = \psi\left(\frac{1}{2}\right) - \psi\left(\frac{1}{2} + \frac{g^2\mu_B^2 B_{c2,\parallel}^2}{2\hbar\tau_{SO}^{-1}} \cdot \frac{1}{2\pi k_B T_c}\right).$$

Here, τ_{SO} is the spin-orbit scattering time, g is the electronic g-factor and μ_B is the Bohr magneton. By fitting this expression to the experimental data of device D2, we obtain a τ_{SO} of ~170 fs for an electron density of 2.38×10^{13} cm^{-2} (Fig. S12A) and ~120 fs for a hole density of -1.10×10^{13} cm^{-2} (Fig. S12B). To verify the applicability of the KLB model, it is important to confirm that the transport scattering time τ_{tr} is smaller than τ_{so}. The transport scattering time can be estimated from the Drude model using the expression $\tau_{tr} = l_{mfp}/v_F$. Here, the Fermi velocity $v_F = \hbar k_F/m^* = \frac{\hbar}{m^*}\sqrt{\frac{4\pi n_e}{g_s g_v}}$. The effective mass m^* is taken as $0.37m_e$ [5]. We obtain a transport scattering time τ_{tr} of ~20 fs for the electron case illustrated in Fig. S12A and ~60 fs for the hole case in Fig. S12B. Since $\tau_{tr} < \tau_{SO}$, the KLB model is indeed applicable and spin-orbit scattering may also contribute to an enhancement of the in-plane critical field, in addition to the increase associated with a strengthening of the superconducting gap due to a pair interaction enhancement as discussed in the main text and section S12 (see below).

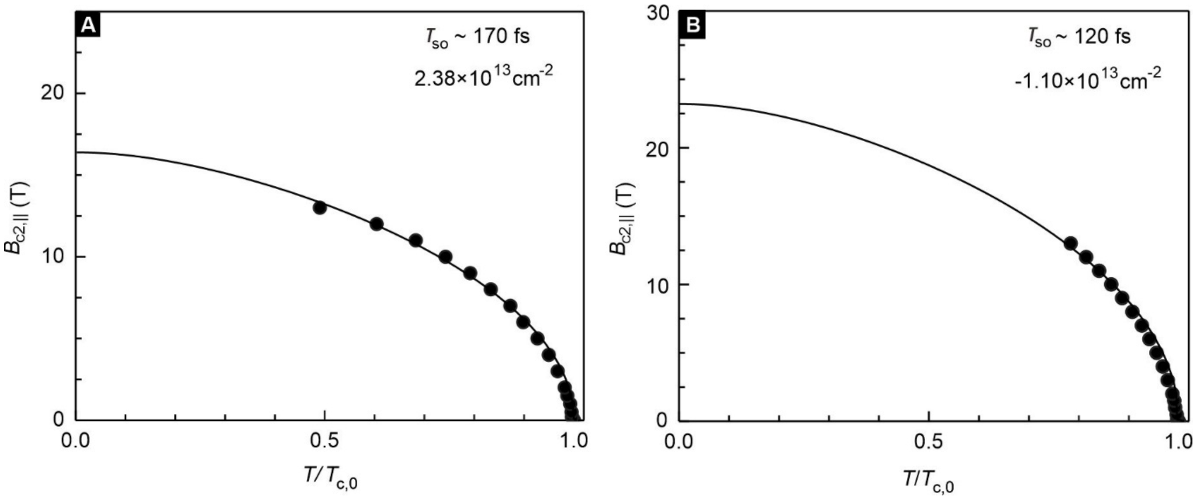

Fig. S12 | Fit of the KLB model to the $B_{c2,\parallel} - T_c$ data of device D2. (A) $B_{c2,\parallel}$ as a function of temperature (circular data points) for an electron density of 2.38×10^{13} cm^{-2}. The solid black line is the fit of the KLB model. **(B)** The same as **(A)** but for a hole density of -1.10×10^{13} cm^{-2}.

11. Magnetic field induced superconductor-to-metal transition

In Fig. S13A, a superconductor-to-metal transition is observed in device D2 at an electron density of 2.60×10^{13} cm^{-2} when applying a perpendicular magnetic field larger than ~3 T. This transition is also confirmed in the magnetic field dependence of the differential resistance shown in Fig. S13B. A dip at low dc bias current changes into a peak near ~3 T. Fig. S13C illustrates the magnetoresistance for different temperatures from 1.65 K to 9 K. The isotherms show crossover points (B_c) within a small magnetic field region near ~3 T. The inset in Fig. S13C shows a close-up view of this crossover area. The width of the crossover region changes with density as illustrated in Fig. S14. It shrinks with increasing density. This observation suggests a magnetic field induced quantum phase transition in this 2D superconductor.

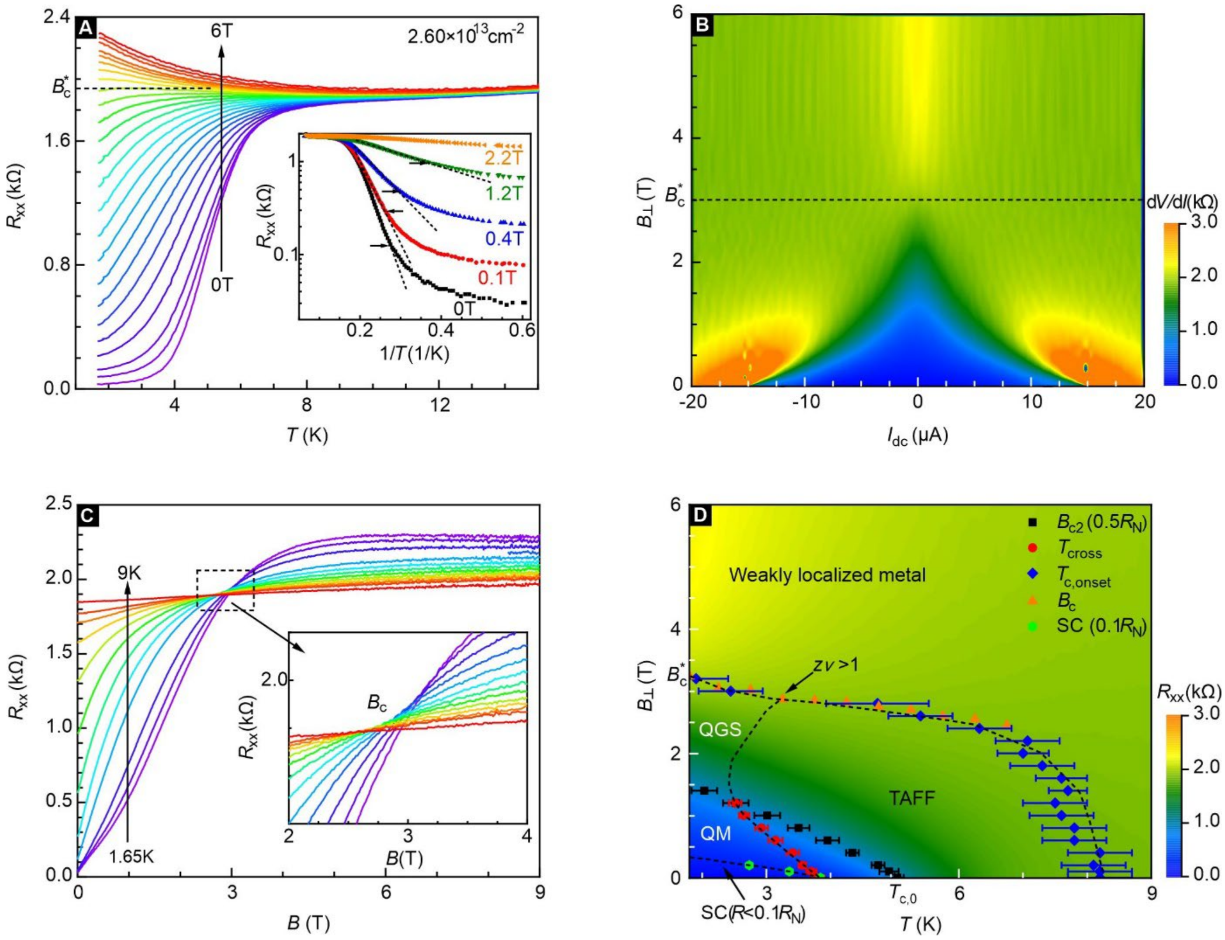

Fig. S13 | Magnetic field driven quantum phase transition in the superconducting regime. All data were taken on device D2 in the superconducting state for an electron density of 2.60×10^{13} cm^{-2}. **(A)** Temperature-dependent resistance at different perpendicular magnetic fields. The inset shows Arrhenius plots of the resistance to highlight the thermally activated behavior. The arrows mark the transition from thermally activated flux flow to a quantum metal state. **(B)** dV/dI versus dc bias current at different magnetic fields at 1.65 K. **(C)** The

magnetoresistance curves at various temperatures from 1.65 to 9 K. The inset shows the crossover region. **(D)** *B*-*T* phase diagram for the following regimes: weakly localized metal, quantum Griffiths state (QGS), thermally activated flux flow (TAFF) and quantum metal. The dashed lines and symbols mark phase boundaries. The different symbols correspond to different criteria to identify the various phases. They are described in more detail in the text.

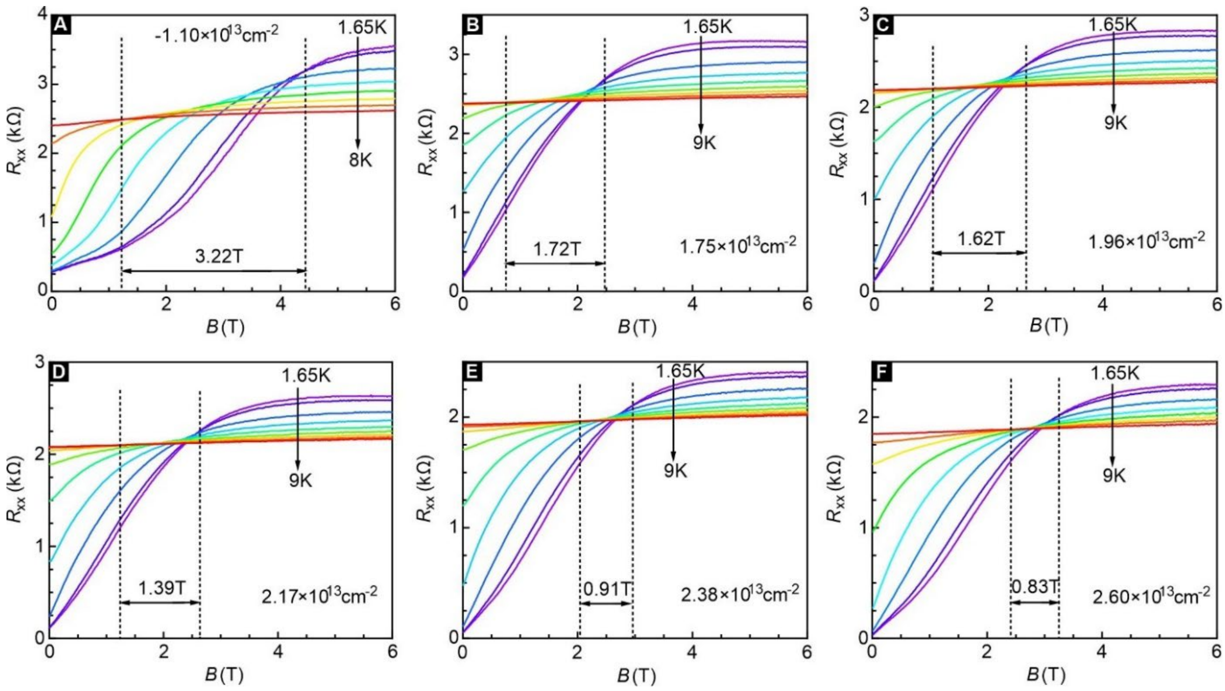

Fig. S14 | Gate dependence of the magnetoresistance in the crossover region at different charge carrier densities. (A-F) Temperature-dependent magnetoresistance recorded on device D2 for temperatures ranging from 1.65 K to 9 K and for a hole density of $-1.10 \times 10^{13}\mathrm{cm}^{-2}$ (**A**) as well as electron densities of $1.75 \times 10^{13}\mathrm{cm}^{-2}$ (**B**), $1.96 \times 10^{13}\mathrm{cm}^{-2}$ (**C**), $2.17 \times 10^{13}\mathrm{cm}^{-2}$ (**D**), $2.38 \times 10^{13}\mathrm{cm}^{-2}$ (**E**) and $2.60 \times 10^{13}\mathrm{cm}^{-2}$ (**F**). The dashed lines in each panel mark the approximate width of the crossover region.

In 2D superconducting systems, fluctuations, disorder, and finite size effects play a particularly important role. They can have a strong impact on the BKT transition, the superconductor-insulator and superconductor-metal transition. For conventional phase transitions, the dynamical critical exponent z only exhibits a singularity at the quantum critical point of the phase diagram itself, while it remains constant near the quantum critical point.

However, in some cases a collection of rare ordered regions can completely destroy the sharpness of the phase transition and this may be the origin of the appearance of a crossover region rather than a single crossover point in Fig. S14. This is reminiscent of a Griffiths singularity. Such a singularity is frequently observed for weakly disordered quantum phase transitions [24,25]. It has been well studied for 3D systems and more recently multiple experimental observations in 2D crystalline superconductors with weak pinning potentials were reported [26–28]. In these studies, multiple critical points were observed and through a finite size scaling analysis, a divergent behavior of the dynamical critical exponent, as expected for a Griffiths singularity, was confirmed. The exponent follows from the power law $z\nu \sim C(B_c^* - B)^{-0.6}$, where C is a constant, B_c^* is the infinite randomness critical point and ν is the static critical exponent. The theoretically predicted value for ν for a superconductor to metal transition is 0.5 [28]. The divergence implies activated scaling with a continuously varying dynamical critical exponent when approaching the infinite-randomness quantum critical point. In our samples, when increasing the charge carrier density, the 2D superconductor becomes more homogeneous and more robust against fluctuations and disorder. This is likely responsible for the shrinking of the crossover region. Fig. S15 summarizes a typical scaling analysis. In order to extract the dynamical critical exponent z, a set of R versus B curves were recorded at different temperatures from 1.7 K to 7.5 K on device D2 for an electron density of 2.60×10^{13} cm^{-2}. There are multiple crossover points (B_c). The finite size scaling law for the isotherms for a proximate crossover point can be expressed as $R(B,T) = R_c \cdot F(|B - B_c|(T/T_0)^{-1/z\nu})$, where F is an arbitrary function obeying the condition that $F(0) = 1$. R_c and B_c are the critical resistance and critical magnetic field, respectively. T_0 is the lowest temperature for the R vs, B curves used in the scaling analysis. By adjusting the value of $z\nu$, curves for different temperatures can be made to collapse onto a single curve [7,28]. An example of this analysis for a narrow range of temperatures between 1.7 K and 1.9 K is shown in Fig. S15B. Fig. S15C plots a quantity that quantifies how well the curves collapse onto each other for a given $z\nu$. The minimum corresponds to the $z\nu$ value that fits best. Fig. S15D displays the optimized value of $z\nu$ as a function of the magnetic field and indeed $z\nu$ rises rapidly with increasing field consistent with divergent behavior. The corresponding change with temperature is included as an insert in Fig. S15C. The data points of Fig. S15D match best the divergent power-law relation $z\nu \sim C(B_c^* - B)^{-0.6}$ and this yields a $B_c^* \sim 3.23$ T. These results provide evidence for a Griffiths singularity at $B = B_c^*$.

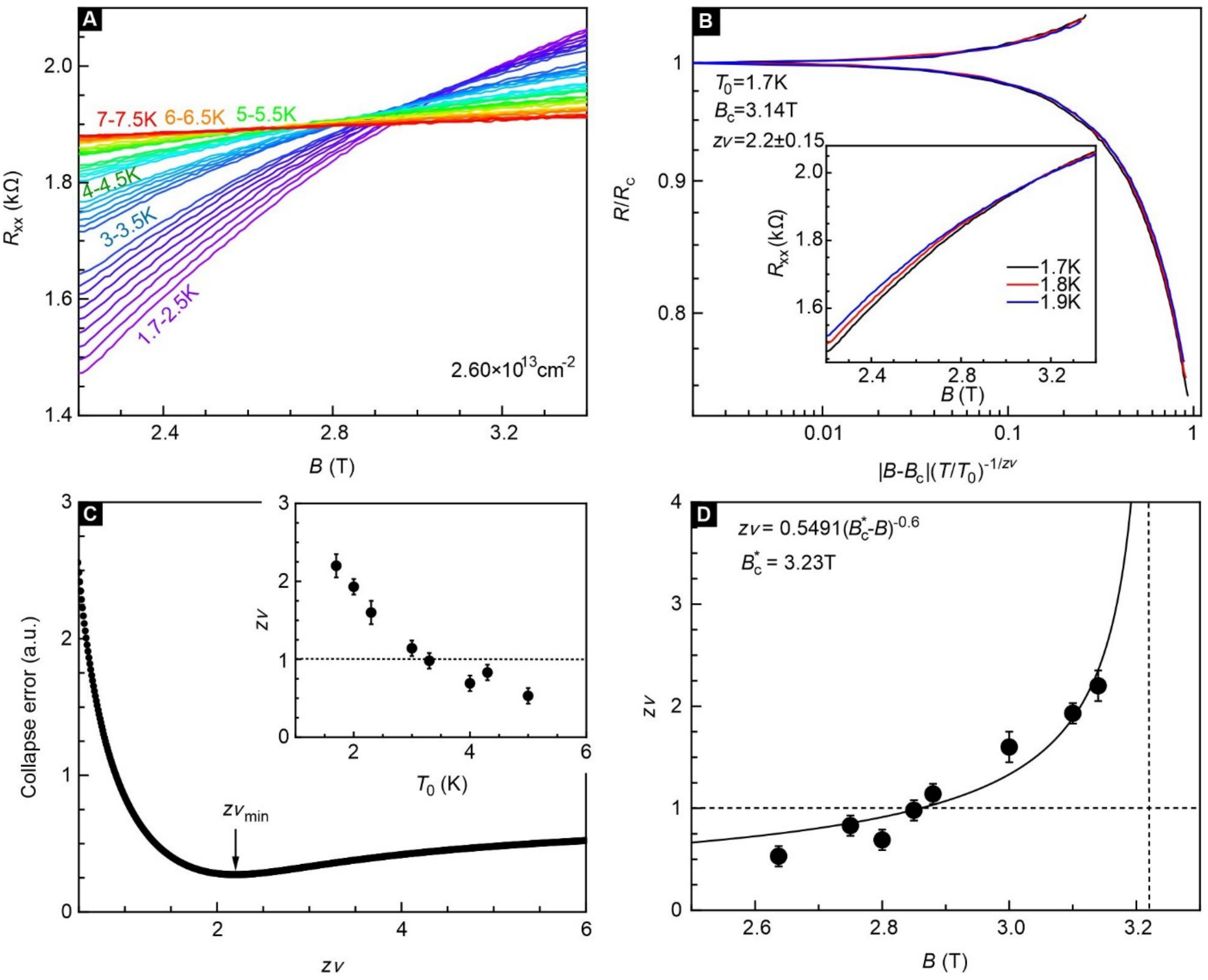

Fig. S15 | Scaling analysis in the context of a quantum Griffiths singularity. (A) A close-up view of multiple crossover points for the R versus B curves in the temperature interval from 1.7 K to 7.5 K. These data sets were recorded on device D2 for an electron density of 2.60×10^{13} cm^{-2}. **(B)** Scaling analysis of the isotherms for the temperature interval between 1.7 K and 2.0 K using the relationship $R/R_c \sim |B - B_c|(T/T_0)^{-1/z\nu}$. Here, T_0 refers to the lowest temperature. It equals 1.7 K. The crossover point is taken as $B_c = 3.14$ T. The inset shows some exemplary R versus B curves in this temperature range. **(C)** The "collapse error" as a function of $z\nu$ to identify the best fit value for $z\nu$. The inset shows the fitted $z\nu$ as a function of temperature. **(D)** Divergent behavior of $z\nu$ as a function of magnetic field B. The solid curve is a fit based on the activation scaling law, $z\nu \sim 0.5491(B_c^* - B)^{-0.6}$. The horizontal dashed line marks $z\nu = 1$ and the vertical line corresponds to $B_c^* = 3.23$ T.

Finally, in Fig. S13D we show a preliminary phase diagram for the transition of the superconducting ground state to the metallic state. Five different criteria are used to determine phase boundaries. The black squares correspond to the out-of-plane upper critical field, $B_{c2,\perp}(T)$. It is defined here as the field where the resistance has dropped to 50% of the normal state resistance using the data in Fig. 13A. The red circles mark the transition from the regime

of thermally activated flux flow (TAFF) to the quantum metal (QM) regime. In experiment this transition is signaled by a deviation from thermally activated behavior as described by the Arrhenius expression. This transition has been marked with arrows in the inset of Fig. 13A. The blue diamonds mark the onset of superconductivity ($T_{c,\text{onset}}$). The orange triangles mark the crossover point of the R versus B curves recorded at neighboring temperatures (see the inset of Fig. S13C). Green hexagons signal the transition to the true superconducting state when $R < 0.1R_N$ and $T < T_{BKT}$ at low magnetic field. With increasing magnetic field, the true superconducting state converts to a weakly localized metallic state via a quantum metal state and/or a quantum Griffith state.

12. Point contact spectroscopy

In this section, we describe in some detail the point contact spectroscopy method deployed here and the results obtained on device D5. Point contact spectroscopy is a technique commonly used to extract information about the energy gap in a superconductor or also a pseudogap. It involves a measurement of either the contact resistance ($R_{contact,dc}$, defined as $R_{contact} = V_{ac}/I_{ac}$ when $I_{dc} = 0$ A) or the differential conductance ($G_{contact} = I_{ac}/V_{ac}$, see Fig. 4 for measuring geometry) across the contact junction as a function of the dc voltage (V_{dc}) for different temperatures and magnetic fields. Here a three terminal measurement configuration is used. A bias current is passed through one of the voltage leads of the device (see Fig. 4 in the main text) and a current lead. We will refer to these leads as contact #1 and contact #2. To impose the bias current a voltage difference must be developed across these two contacts and it is composed of three distinct contributions: a voltage drop ΔV_1 across the junction between voltage lead #1 and the $MoTe_2$ layer, ΔV_{MoTe2} due to the resistance of the $MoTe_2$ layer itself and ΔV_2 due to the junction between the $MoTe_2$ layer and the current lead #2. By measuring the voltage drop between voltage lead #1 and a second voltage lead (#3), that does not carry and current, we only measure ΔV_1, the quantity of interest. The voltage drop across a small portion of the $MoTe_2$ should be of no concern, since $MoTe_2$ remains in the superconducting state during these point-contact measurements (see Fig. S16B). Hence, we can unambiguously obtain the voltage drop across the junction formed by voltage lead #1 and the $MoTe_2$ layer and there is no risk of overestimating the gap size in the selected geometry. The same approach is followed to measure the differential resistance at fixed bias across the junction.

The quality of the contact area is of great importance for point contact spectroscopy. Here in this work, the point contact is formed by placing part of the thin film on top of a micron-

sized Au electrode. The contact area is determined by the grain size of the sputtered Au electrodes, and it is inevitable that many parallel channels co-exist. Without exerting any additional pressure, the contact is of the van der Waals type [29–31]. Since multiple channels contribute in parallel, the recorded differential conductance represents a spatial average. The superconducting gap appears as a bump like structure in the differential conductance versus bias voltage when the sample is cooled below the critical temperature. The bump becomes stronger at the temperature is lowered and develops a center dip flanked by the two coherence peaks as seen in panel C and E of Fig. 4 in the main text. The recorded $G_{contact}$ traces typically exhibit such a clear gap signature with a pronounced temperature and magnetic field dependence. We note that the point contact spectroscopy is performed with the already available leads on the sample. Contacts that fulfill the necessary conditions are selected after sample fabrication and basic characterization.

By combining the point contact spectroscopy results with in-plane resistance measurements on the same sample, it is possible to exclude extrinsic or thermal effects as the origin of the feature observed in the point contact spectroscopy. Fig. S16A plots the temperature dependence of the resistance for contact #1 ($R_{contact,dc}$, black line) together with the in-plane sheet resistance (R_{sheet}, blue line). These data were taken simultaneously. The dashed line marks the transition temperature $T_{c,\mathrm{onset}}$ of ~7.5K. The four terminal sheet resistance exhibits the typical superconducting transition and it drops down to zero. The contact resistance keeps increasing before it turns down near 3K. A small kink near 7-8K agrees well with the superconducting transition point in R_{sheet}. Fig. S16B presents the corresponding differential resistance data as a function of the dc bias current I_{dc}: $R_{contact}$ for contact #1 as a black line and the in-plane differential resistance $\mathrm{d}V/\mathrm{d}I_{\mathrm{sheet}}$ in blue. A single peak is observed in $R_{contact}$ near zero I_{dc}, while $\mathrm{d}V/\mathrm{d}I_{\mathrm{sheet}}$ remains constant and close to zero within the explored current range of 2μA. The latter demonstrates that the sample remains in the superconducting ground state within this range of bias currents. If we assume the behavior of $R_{contact}$ with bias current in Fig. S16B is the result of a bias current related thermal effect, we would anticipate similar behavior in the temperature dependence of $R_{contact,dc}$ shown in Fig. S16A. However, both curves are qualitatively very different and hence thermal effects can be excluded.

The evolution of the differential conductance $G_{contact}$ of contact #1 with an applied perpendicular magnetic field is shown in Fig. S16C. The magnetic field varies from 0 to 6 T. The temperature dependence can be found in Fig. 4C of the main text. To extract information about the superconducting gap, the extended Blonder–Tinkham–Klapwijk (BTK) model can

be used to fit the $G_{contact}$ data [29–32]. From the present point contact spectroscopy data, it is difficult to determine the symmetry of the superconducting gap, i.e. whether we are dealing with isotropic s-wave, or nodal-like pairing such as anisotropic s-wave, p-wave or d-wave pairing. Previous results obtained on bulk materials provided strong evidence for unconventional $s^{\pm}$-wave pairing [33,34]. We therefore resort to an extended single-band isotropic s-wave BTK model to fit the normalized $G_{contact}$ data in Fig. S16D. The normalization of the $G_{contact}$ data proceeds, as usual, by subtracting data obtained at some temperature above the superconducting transition temperature. Here, either data at 8K or 15K were used for this purpose. These data sets are vertically shifted until the data points at higher bias voltage match with those of the low-temperature data. Subsequently, the shifted data sets are used to normalize the low temperature data by division. Examples of normalized data traces are shown in Fig. S16D. The fit procedure yields the following parameters: the gap size Δ, the parameter describing the potential barrier Z, and a phenomenological broadening factor Γ. Fits to the data traces using the BKT model yield the following approximate gap values: 3.0 meV (left panel) and 3.1 meV (right panel) using 15K and 8K data for normalization respectively). This result confirms the validity of the normalization procedure as there is no significant influence of the high temperature data set used. The temperature dependence of the differential conductance $G_{contact}$ of contact #1 plotted in Fig. 4B suggests that the contact is in the insulating regime. The feature associated with a gap persists up to temperatures much higher than $T_{c,\mathrm{onset}}$ and in Fig 4D there is a deviation from the BCS relation. Such behavior is unanticipated and consistent with pseudogap behavior. In contrast, the conductance data for contact #2 in Fig. 4E does not exhibit such pseudogap behavior. For this data set we are in the metallic regime and we observe Andreev reflection and enhanced conductance below the superconducting gap. These two different behaviors in the insulating and metallic regime are not well understood. It possibly reflects some extrinsic effect such as a gap distribution over a wide energy range due to spatial inhomogeneities [35]. The fitted gap amplitudes are summarized in Fig. 4D and F for both contacts. The phenomenological broadening parameter Γ is shown in Fig. S16E for contact #1 and #2. We note that extrinsic effects are "absorbed" in the phenomenological broadening parameter Γ in this BTK fitting, so that they have little influence on the gap size. For the sake of completeness, we note that the pseudogap behavior has been reported in a number of previous studies, such as low-density superconductors [36] and cuprates [37].

Fig. S17 presents point-contact data at different temperatures and magnetic fields both in the insulating regime at a low hole density of -2.2×10^{12} cm^{-2} and in the superconducting

regime for a hole density of -1.07×10^{13} cm^{-2}. Gap-like behavior is observed even in the insulating regime. This is not anticipated at first. However, as a result of the spatial inhomogeneity superconducting puddles may exist in the contact area even in the insulating states and produce superconducting gap behavior with a reduced gap value. In these two regimes, the above normalization method brings large uncertainty when performing the BTK fittings, as the spectra at higher bias voltage deviate a lot from 1. Nonetheless, rough estimation of the gap size yields the gap values of 2.3 ± 0.2 meV and 1.8 ± 0.2 meV for insulating and hole-doped superconducting regime respectively, as marked with dashed black lines in Fig. S18. The corresponded gap ratios ($\Delta/k_{\mathrm{B}}\,T_{c,\mathrm{onset}}$) are 3.5 and 2.8 respectively, hence both exceed the BCS limit, supporting the strong pairing interaction in this hole-doped superconducting regime as well.

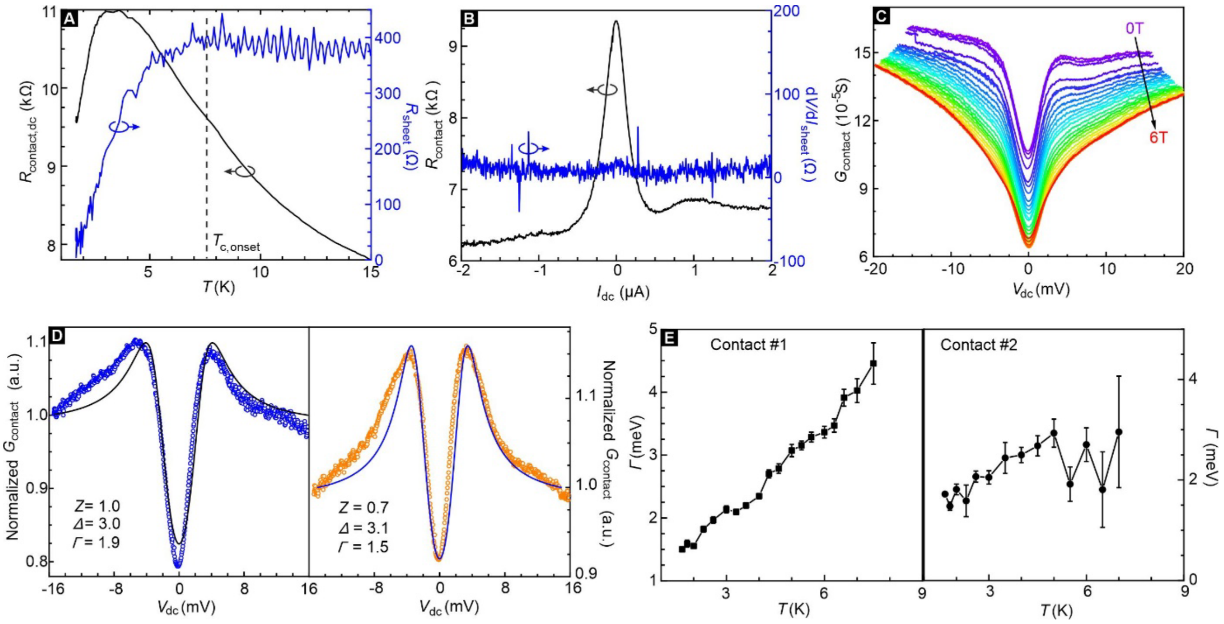

Fig. S16 | Analysis of the point-contact spectroscopy data. All data shown were recorded on device D5 for an electron density of 1.38×10^{13} cm^{-2}. **(A)** Temperature dependence of the contact resistance $R_{contact,dc}$ for contact #1 (black line) and the in-plane sheet resistance R_{sheet} (blue line). The dashed line marks the onset of the superconducting transition ($T_{c,\mathrm{onset}}$). **(B)** Differential resistance $R_{contact}$ for contact #1 (black line) and the in-plane differential sheet resistance dV/dI_{sheet} (blue line) as a function of the bias current I_{dc}. **(C)** Differential conductance $G_{contact}$ as a function of the dc voltage V_{dc} for different values of the magnetic field from 0T to 6 T in 0.5 T steps. Data are recorded on contact #1 at a fixed temperature of 1.65 K. **(D)** Fit of the BTK model to the differential conductance data recorded at 1.65 K for contact #1. The data is either normalized using 15 K (left panel) or 8 K (right panel) high temperature data. **(E)** Temperature dependence of the broadening parameter Γ extracted from the BTK fit. Data are shown for both contact #1 (left panel) and contact #2 (right panel).

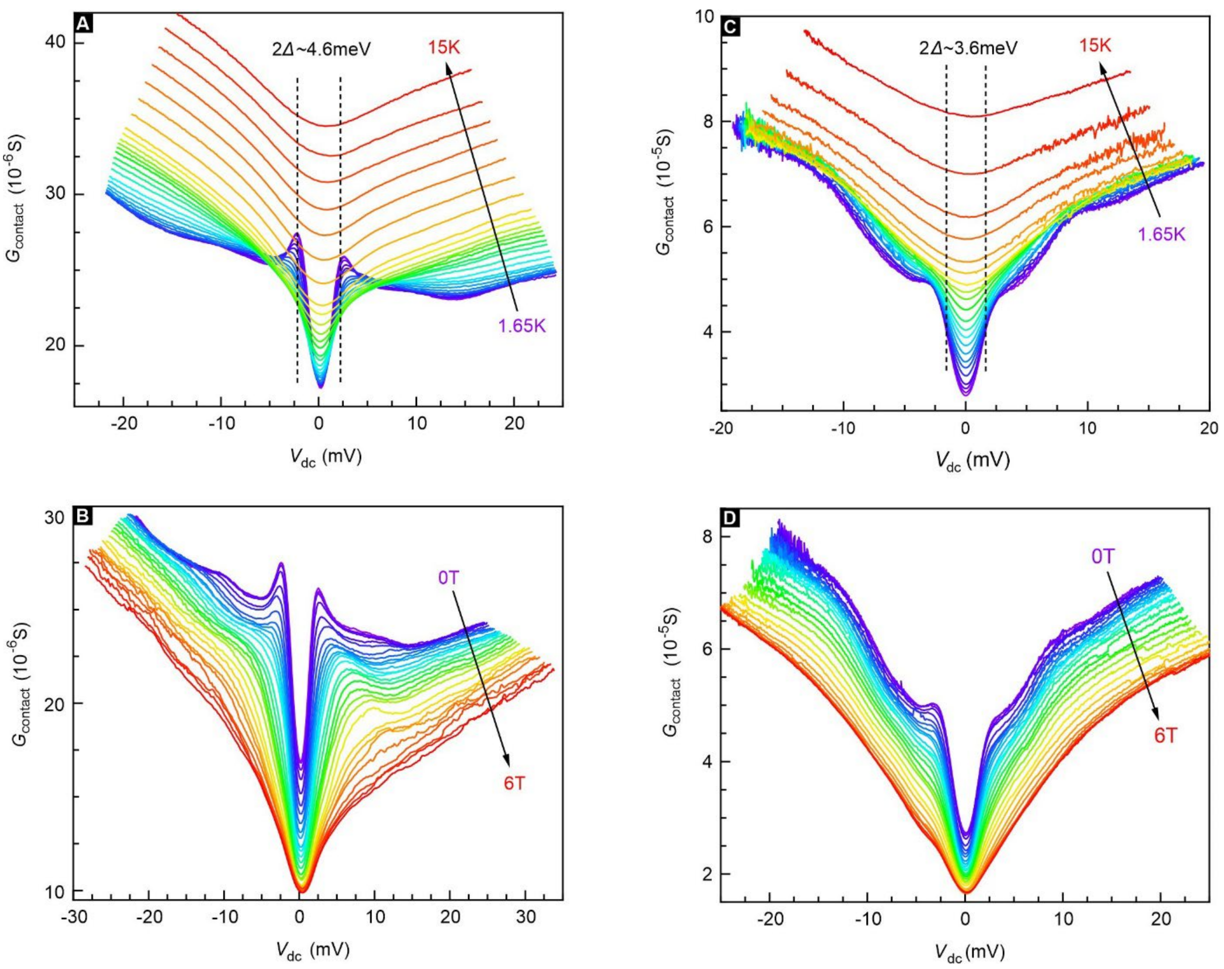

Fig. S17 | Point-contact spectroscopy in the insulating and hole-doped superconducting regime. Data were recorded on contact #1 of device D5**.** **(A)** Temperature evolution of the differential conductance $G_{contact}$ recorded as a function of V_{dc} for B = 0 T and a low net hole density of -2.2×10^{12} cm^{-2}. The temperature is varied from 1.65K to 15K. The dashed lines demarcate the gap region. **(B)** Differential conductance $G_{contact}$ as in panel **A** but for different values of the perpendicular magnetic field (0T to 6T) at a fixed temperature of 1.65 K. **(C** and **D)** Same as (**A** and **B)** but for a hole density of -1.07×10^{13} cm^{-2}, respectively.

13. Monolayer device showing no evidence of a bulk gap

The monolayer devices that were fabricated in an argon glove box environment and encapsulated immediately after exfoliation and placement on the prepatterned substrate showed insulating behavior. We attribute this to the opening of a gap. Here we wish to document the data obtained on a monolayer device with different transport behavior (device D6) from all other measured monolayer samples in this work. It exhibits superconductivity for the full range of gate voltages without any sign for the emergence of a bulk gap. This sample does not exhibit the onset of superconductivity at a higher temperature than the other monolayer devices and,

hence, we conclude that the superconductivity is not enhanced compared with the insulating monolayer devices. The disorder induced density in this sample is on the order of 10^{12} cm^{-2}. The behavior of this sample resembles what has been reported previously in the literature[5]. While this sample was fabricated using the same processing steps as all other samples, it was left on the Si substrate surface for approximately 5 days prior to protective encapsulation with a thin hBN flake and subsequent transfer onto the substrate with pre-patterned electrodes. Even though the sample was stored inside the argon atmosphere of the glove box, sample degradation may have occurred during this long delay between exfoliation and encapsulation. Partial oxidation may increase the doping and be responsible for the distinct behavior of this sample as compared to the other monolayer samples that were protected by encapsulated immediately. Fig. S18A displays an optical image of the device. In panel B the band structure schematic for a semi-metal is shown that appears to be applicable for this device. Transport results are shown in this figure as well: R versus T (C), R versus B (D) and the differential resistance dV/dI as a function of the dc bias current I_{dc} (E). In all cases, the charge carrier density serves as an additional parameter. The thick black trace in Fig. S18C corresponds to average zero carrier density using the criterion that at the applied gate voltages, the resistance exhibits a peak in the normal state at 10 K. At this gate voltage condition the critical current also reaches its minimum (Fig. S18E). Fig. S18F plots the temperature at the transition of superconductivity $T_{c,0}$ as well as the critical current I_c as a function of the charge carrier density.

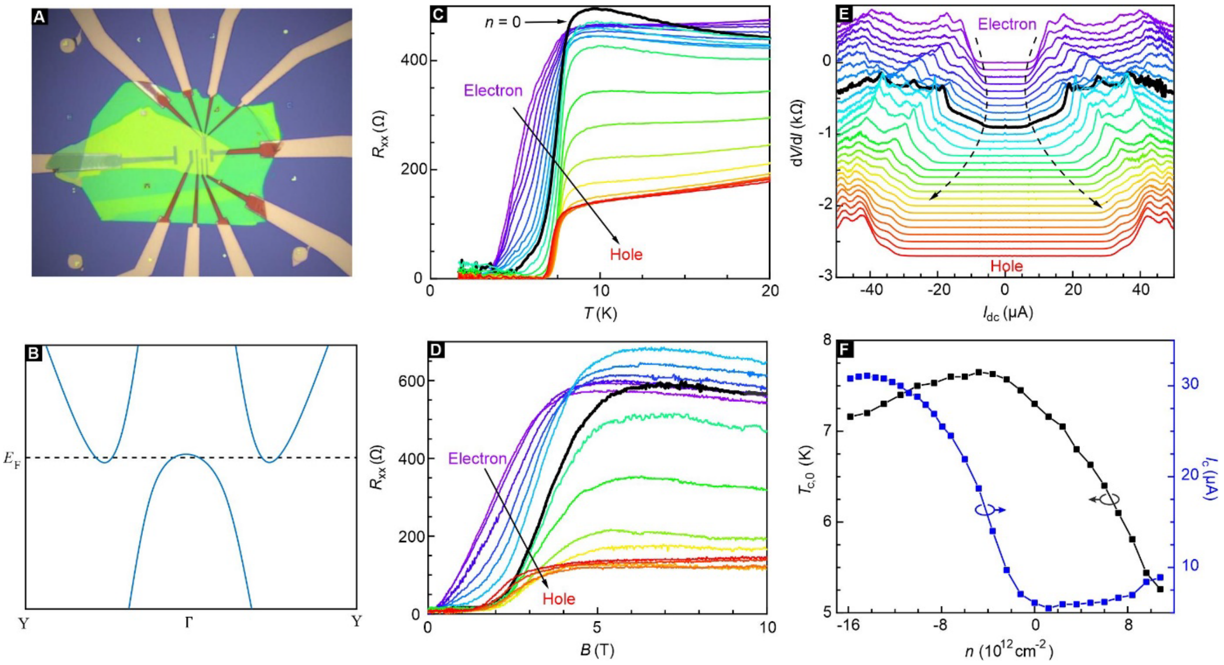

Fig. S18 | Monolayer device showing no evidence of a bulk gap (device D6). **(A)** Optical image of the device. **(B)** Schematic band structure for a semi-metal as applicable to this device.

(C) Temperature dependence of the sheet resistance for different carrier densities. Data are recorded in the absence of a magnetic field. The thick black line is recorded at gate voltages corresponding to zero average density. **(D)** Sheet resistance as a function of the perpendicular magnetic field. The charge carrier density serves as an additional parameter. All data taken at 1.65 K. **(E)** Differential resistance as a function of the dc-bias current. The charge carrier density serves as an additional parameter. These data are used to extract the critical current I_c in panel **F**. Data are recorded at T = 1.65 K and B = 0 T. **(F)** Temperature at the transition of superconductivity, $T_{c,0}$, and critical current, I_c, as a function of the average carrier density. The density range in panel **C-F** is $-1.51 \times 10^{13} < n < 1.08 \times 10^{13}$ cm^{-2}.

14. Comparison with other 2D superconductors

Key parameters of our superconducting samples are compared with other 2D superconductors in the table below.

2D SC	$T_{c,onset}$ (K)	n (cm^{-2})	Gate range (cm^{-2})	$B_{c2,\perp}$ (T)	$B_{c2,\parallel}$ (T)	Hole or electron SC	Inversion symmetry	Out of plane symmetry
**(type-I)** 2H-$NbSe_2$[38]	~3.2	>10^{14}	Not applicable	~1	>30 (~6B_p)	hole	no	yes
**(type-I)** 2H-TaS_2[39]	~2.2	>10^{14}	Not applicable	~1	>30 (~6B_p)	Not known	no	yes
**(type-I)** 2H-MoS_2[40]	~10	~7×10^{13}	liquid gate ~10^{14}	0.5	>50 (~6B_p)	electron	no	yes
**(type-II)** Stanene[41]	~1.5	>10^{14}	Not applicable	~0.6	~4 (~4B_p)	two hole bands	yes	no
**(type-II)** $PdTe_2$[42]	~0.7	>10^{14}	Not applicable	~0.8	~20 (~7B_p)	two bands	yes	no
TBLG[43]	~2	~1.4×10^{12}	2×10^{13}	~0.1	~1 ~1B_p)	ambipolar	yes	no
TTLG[44]	~3	~3×10^{12}	2×10^{13}	~0.4	~10 (~3B_p)	ambipolar	no	yes
1T'-WTe_2 [6,7]	~1.2	~5×10^{12}	2×10^{13}	~0.05	~4.5 (~6B_p)	electron	yes	no
**1T'-$MoTe_2$**	**~7.5**	**~5×10^{12}**	**2×10^{13}**	**~2**	**~20 (~2B_p)**	**ambipolar**	**yes**	**no**

Table S1. Comparison of key parameters of the superconducting 1T'-$MoTe_2$ monolayers with some other 2D superconductors, that have been reported in the literature. They include type-I and type-II Ising superconductors, twisted graphene layers (TBLG = twisted bilayer graphene, TTLG = twisted trilayer graphene) and monolayer 1T'-WTe_2. In some cases the density is not known.

15. References

(1) Gan, Y.; Cho, C.-W.; Li, A.; Lyu, J.; Du, X.; Wen, J.-S.; Zhang, L.-Y. Giant Enhancement of Superconductivity in Few Layers $MoTe_2$. *Chinese Phys. B* **2019**, *28* (11), 117401.

(2) Keum, D. H.; Cho, S.; Kim, J. H.; Choe, D. H.; Sung, H. J.; Kan, M.; Kang, H.; Hwang, J. Y.; Kim, S. W.; Yang, H.; Chang, K. J.; Lee, Y. H. Bandgap Opening in Few-Layered Monoclinic $MoTe_2$. *Nat. Phys.* **2015**, *11* (6), 482–486.

(3) Kim, Y.; Balram, A. C.; Taniguchi, T.; Watanabe, K.; Jain, J. K.; Smet, J. H. Even Denominator Fractional Quantum Hall States in Higher Landau Levels of Graphene. *Nat. Phys.* **2019**, *15* (2), 154–158.

(4) Cheon, Y.; Lim, S. Y.; Kim, K.; Cheong, H. Structural Phase Transition and Interlayer Coupling in Few-Layer 1T′ and T_d $MoTe_2$. *ACS Nano* **2021**, *15* (2), 2962–2970.

(5) Rhodes, D. A.; Jindal, A.; Yuan, N. F. Q.; Jung, Y.; Antony, A.; Wang, H.; Kim, B.; Chiu, Y.; Taniguchi, T.; Watanabe, K.; Barmak, K.; Balicas, L.; Dean, C. R.; Qian, X.; Fu, L.; Pasupathy, A. N.; Hone, J. Enhanced Superconductivity in Monolayer T_d-$MoTe_2$. *Nano Lett.* **2021**, *21* (6), 2505–2511.

(6) Fatemi, V.; Wu, S.; Cao, Y.; Bretheau, L.; Gibson, Q. D.; Watanabe, K.; Taniguchi, T.; Cava, R. J.; Jarillo-Herrero, P. Electrically Tunable Low-Density Superconductivity in a Monolayer Topological Insulator. *Science* **2018**, *362* (6417), 926–929.

(7) Sajadi, E.; Palomaki, T.; Fei, Z.; Zhao, W.; Bement, P.; Olsen, C.; Luescher, S.; Xu, X.; Folk, J. A.; Cobden, D. H. Gate-Induced Superconductivity in a Monolayer Topological Insulator. *Science* **2018**, *362* (6417), 922–925.

(8) Tang, S.; Zhang, C.; Jia, C.; Ryu, H.; Hwang, C.; Hashimoto, M.; Lu, D.; Liu, Z.; Devereaux, T. P.; Shen, Z. X.; Mo, S. K. Electronic Structure of Monolayer 1T′-$MoTe_2$ Grown by Molecular Beam Epitaxy. *APL Mater.* **2018**, *6* (2), 026601.

(9) Sun, B.; Zhao, W.; Palomaki, T.; Fei, Z.; Runburg, E.; Malinowski, P.; Huang, X.; Cenker, J.; Cui, Y. T.; Chu, J. H.; Xu, X.; Ataei, S. S.; Varsano, D.; Palummo, M.; Molinari, E.; Rontani, M.; Cobden, D. H. Evidence for Equilibrium Exciton Condensation in Monolayer WTe_2. *Nat. Phys.* **2022**, *18* (1), 94–99.

(10) Jia, Y.; Wang, P.; Chiu, C. L.; Song, Z.; Yu, G.; Jäck, B.; Lei, S.; Klemenz, S.; Cevallos, F. A.; Onyszczak, M.; Fishchenko, N.; Liu, X.; Farahi, G.; Xie, F.; Xu, Y.; Watanabe, K.; Taniguchi, T.; Bernevig, B. A.; Cava, R. J.; Schoop, L. M.; Yazdani, A.; Wu, S. Evidence for a Monolayer Excitonic Insulator. *Nat. Phys.* **2022**, *18* (1), 87–93.

(11) Gan, Y.; Liang, J.; Cho, C. woo; Li, S.; Guo, Y.; Ma, X.; Wu, X.; Wen, J.; Du, X.; He, M.; Liu, C.; Yang, S. A.; Wang, K.; Zhang, L. Bandgap Opening in $MoTe_2$ Thin Flakes Induced by Surface Oxidation. *Front. Phys.* **2020**, *15* (3), 1–7.

(12) Tang, F.; Wang, P.; He, M.; Isobe, M.; Gu, G.; Li, Q.; Zhang, L.; Smet, J. H. Two-Dimensional Quantum Hall Effect and Zero Energy State in Few-Layer $ZrTe_5$. *Nano Lett.* **2021**, *21* (14), 5998–6004.

(13) Cao, Y.; Luo, J. Y.; Fatemi, V.; Fang, S.; Sanchez-Yamagishi, J. D.; Watanabe, K.; Taniguchi, T.; Kaxiras, E.; Jarillo-Herrero, P. Superlattice-Induced Insulating States and Valley-Protected Orbits in Twisted Bilayer Graphene. *Phys. Rev. Lett.* **2016**, *117* (11), 1–5.

(14) Roth, A.; Brune, C.; Buhmann, H.; Molenkamp, L. W.; Maciejko, J.; Qi, X.-L.; Zhang, S.-C. Nonlocal Transport in the Quantum Spin Hall State. *Science* **2009**, *325* (5938), 294–297.

(15) Konig, M.; Wiedmann, S.; Brune, C.; Roth, A.; Buhmann, H.; Molenkamp, L. W.; Qi, X.-L.; Zhang, S.-C. Quantum Spin Hall Insulator State in HgTe Quantum Wells. *Science* **2007**, *318* (5851), 766–770.

(16) Wu, S.; Fatemi, V.; Gibson, Q. D.; Watanabe, K.; Taniguchi, T.; Cava, R. J.; Jarillo-Herrero, P. Observation of the Quantum Spin Hall Effect up to 100 Kelvin in a Monolayer Crystal. *Science* **2018**, *359* (6371), 76–79.

(17) Fei, Z.; Palomaki, T.; Wu, S.; Zhao, W.; Cai, X.; Sun, B.; Nguyen, P.; Finney, J.; Xu, X.; Cobden, D. H. Edge Conduction in Monolayer WTe_2. *Nat. Phys.* **2017**, *13* (7), 677–682.

(18) Kapitulnik, A.; Kivelson, S. A.; Spivak, B. Colloquium: Anomalous Metals: Failed Superconductors. *Rev. Mod. Phys.* **2019**, *91* (1), 11002.

(19) Jaeger, H. M.; Haviland, D. B.; Orr, B. G.; Goldman, A. M. Onset of Superconductivity in Ultrathin Granular Metal Films. *Phys. Rev. B* **1989**, *40* (1), 182–196.

(20) Kawaguchi, G.; Bardin, A. A.; Suda, M.; Uruichi, M.; Yamamoto, H. M. An Ambipolar Superconducting Field-Effect Transistor Operating above Liquid Helium Temperature. *Adv. Mater.* **2019**, *31* (2), 1–6.

(21) Michael Tinkham. *Introduction to Superconductivity*, 2nd ed.; Dover Publications, Inc., 1996.

(22) Tsen, A. W.; Hunt, B.; Kim, Y. D.; Yuan, Z. J.; Jia, S.; Cava, R. J.; Hone, J.; Kim, P.; Dean, C. R.; Pasupathy, A. N. Nature of the Quantum Metal in a Two-Dimensional Crystalline Superconductor. *Nat. Phys.* **2016**, *12* (3), 208–212.

(23) Klemm, R. A.; Luther, A.; Beasley, M. R. Theory of the Upper Critical Field in Layered Superconductors. *Phys. Rev. B* **1975**, *12* (3), 877–891.

(24) Harris, A. B. Effect of Random Defects on the Critical Behaviour of Ising Models. *J. Phys. C Solid State Phys.* **1974**, *7* (9), 1671–1692.

(25) Vojta, T. Rare Region Effects at Classical, Quantum and Nonequilibrium Phase Transitions. *J. Phys. A. Math. Gen.* **2006**, *39* (22), R143–R205.

(26) Saito, Y.; Nojima, T.; Iwasa, Y. Highly Crystalline 2D Superconductors. *Nat. Rev. Mater.* **2016**, *2* (1), 1–18.

(27) Xing, Y.; Zhang, H. M.; Fu, H. L.; Liu, H.; Sun, Y.; Peng, J. P.; Wang, F.; Lin, X.; Ma, X. C.; Xue, Q. K.; Wang, J.; Xie, X. C. Quantum Griffiths Singularity of Superconductor-Metal Transition in Ga Thin Films. *Science* **2015**, *350* (6260), 542–545.

(28) Saito, Y.; Nojima, T.; Iwasa, Y. Quantum Phase Transitions in Highly Crystalline Two-Dimensional Superconductors. *Nat. Commun.* **2018**, *9* (1), 1–7.

(29) Daghero, D.; Tortello, M.; Ummarino, G. A.; Gonnelli, R. S. Directional Point-Contact Andreev-Reflection Spectroscopy of Fe-Based Superconductors: Fermi Surface Topology, Gap Symmetry, and Electron–Boson Interaction. *Reports Prog. Phys.* **2011**, *74* (12), 124509.

(30) Daghero, D.; Pecchio, P.; Ummarino, G. A.; Nabeshima, F.; Imai, Y.; Maeda, A.; Tsukada, I.; Komiya, S.; Gonnelli, R. S. Point-Contact Andreev-Reflection Spectroscopy in Fe(Te,Se) Films: Multiband Superconductivity and Electron-Boson Coupling. *Supercond. Sci. Technol.* **2014**, *27* (12), 124014.

(31) Tang, F.; Wang, P.; Wang, P.; Gan, Y.; Gu, G. D.; Zhang, W.; He, M.; Zhang, L. Quasi-2D Superconductivity in $FeTe_{0.55}Se_{0.45}$ Ultrathin Film. *J. Phys. Condens. Matter* **2019**, *31* (26), 265702.

(32) Blonder, G. E.; Tinkham, M.; Klapwijk, T. M. Transition from Metallic to Tunneling Regimes in Superconducting Microconstrictions: Excess Current, Charge Imbalance, and Supercurrent Conversion. *Phys. Rev. B* **1982**, *25* (7), 4515–4532.

(33) Li, Y.; Gu, Q.; Chen, C.; Zhang, J.; Liu, Q.; Hu, X.; Liu, J.; Liu, Y.; Ling, L.; Tian, M.; Wang, Y.; Samarth, N.; Li, S.; Zhang, T.; Feng, J.; Wang, J. Nontrivial Superconductivity in Topological $MoTe_{2-x}S_x$ Crystals. *Proc. Natl. Acad. Sci. U. S. A.* **2018**, *115* (38), 9503–9508.

(34) Guguchia, Z.; Von Rohr, F.; Shermadini, Z.; Lee, A. T.; Banerjee, S.; Wieteska, A. R.; Marianetti, C. A.; Frandsen, B. A.; Luetkens, H.; Gong, Z.; Cheung, S. C.; Baines, C.; Shengelaya, A.; Taniashvili, G.; Pasupathy, A. N.; Morenzoni, E.; Billinge, S. J. L.; Amato, A.; Cava, R. J.; Khasanov, R.; Uemura, Y. J. Signatures of the Topological s^{+-} Superconducting Order Parameter in the Type-II Weyl Semimetal T_d-$MoTe_2$. *Nat. Commun.* **2017**, *8* (1), 1–8.

(35) Boschker, H.; Fillis-Tsirakis, E.; Richter, C.; Zhang, D.; Smet, J.; Kuerten, L. Microscopic Origin of the Dynes Parameter Γ of the $LaAlO_3$-$SrTiO_3$ Interface Superconductor. *Phys. Rev. B* **2020**, *102* (13), 1–9.

(36) Nakagawa, Y.; Saito, Y.; Nojima, T.; Inumaru, K.; Yamanaka, S.; Kasahara, Y.; Iwasa, Y. Gate-Controlled Low Carrier Density Superconductors: Toward the Two-Dimensional BCS-BEC Crossover. *Phys. Rev. B* **2018**, *98* (6), 1–8.

(37) Miyakawa, N.; Zasadzinski, J. F.; Ozyuzer, L.; Guptasarma, P.; Hinks, D. G.; Kendziora, C.; Gray, K. E. Predominantly Superconducting Origin of Large Energy Gaps in Underdoped $Bi_2Sr_2CaCu_2O_{8+\delta}$ from Tunneling Spectroscopy. *Phys. Rev. Lett.* **1999**, *83* (5), 1018–1021.

(38) Xi, X.; Wang, Z.; Zhao, W.; Park, J.-H.; Law, K. T.; Berger, H.; Forró, L.; Shan, J.; Mak, K. F. Ising Pairing in Superconducting $NbSe_2$ Atomic Layers. *Nat. Phys.* **2016**, *12* (2), 139–143.

(39) de la Barrera, S. C.; Sinko, M. R.; Gopalan, D. P.; Sivadas, N.; Seyler, K. L.; Watanabe, K.; Taniguchi, T.; Tsen, A. W.; Xu, X.; Xiao, D.; Hunt, B. M. Tuning Ising Superconductivity with Layer and Spin–Orbit Coupling in Two-Dimensional Transition-Metal Dichalcogenides. *Nat. Commun.* **2018**, *9* (1), 1427.

(40) Ye, J. T.; Zhang, Y. J.; Akashi, R.; Bahramy, M. S.; Arita, R.; Iwasa, Y. Superconducting Dome in a Gate-Tuned Band Insulator. *Science* **2012**, *338* (6111), 1193–1196.

(41) Falson, J.; Xu, Y.; Liao, M.; Zang, Y.; Zhu, K.; Wang, C.; Zhang, Z.; Liu, H.; Duan, W.; He, K.; Liu, H.; Smet, J. H.; Zhang, D.; Xue, Q. K. Type-II Ising Pairing in Few-Layer Stanene. *Science* **2020**, *367* (6485), 1454–1457.

(42) Liu, Y.; Xu, Y.; Sun, J.; Liu, C.; Liu, Y.; Wang, C.; Zhang, Z.; Gu, K.; Tang, Y.; Ding, C.; Liu, H.; Yao, H.; Lin, X.; Wang, L.; Xue, Q. K.; Wang, J. Type-II Ising Superconductivity and Anomalous Metallic State in Macro-Size Ambient-Stable Ultrathin Crystalline Films. *Nano Lett.* **2020**, *20* (8), 5728–5734.

(43) Cao, Y.; Fatemi, V.; Fang, S.; Watanabe, K.; Taniguchi, T.; Kaxiras, E.; Jarillo-Herrero, P. Unconventional Superconductivity in Magic-Angle Graphene Superlattices. *Nature* **2018**, *556* (7699), 43–50.

(44) Park, J. M.; Cao, Y.; Watanabe, K.; Taniguchi, T.; Jarillo-Herrero, P. Tunable Strongly Coupled Superconductivity in Magic-Angle Twisted Trilayer Graphene. *Nature* **2021**, *590* (7845), 249–255.
